# Raf1 Is a DCAF for the Rik1 DDB1-Like Protein and Has Separable Roles in siRNA Generation and Chromatin Modification

**DOI:** 10.1371/journal.pgen.1002499

**Published:** 2012-02-02

**Authors:** Alessia Buscaino, Sharon A. White, Douglas R. Houston, Erwan Lejeune, Femke Simmer, Flavia de Lima Alves, Piyush T. Diyora, Takeshi Urano, Elizabeth H. Bayne, Juri Rappsilber, Robin C. Allshire

**Affiliations:** 1 Wellcome Trust Centre for Cell Biology, School of Biological Sciences, The University of Edinburgh, Edinburgh, United Kingdom; 2 Department of Biochemistry, Shimane University Faculty of Medicine, Izumo, Japan; Fred Hutchinson Cancer Research Center, United States of America

## Abstract

Non-coding transcription can trigger histone post-translational modifications forming specialized chromatin. In fission yeast, heterochromatin formation requires RNAi and the histone H3K9 methyltransferase complex CLRC, composed of Clr4, Raf1, Raf2, Cul4, and Rik1. CLRC mediates H3K9 methylation and siRNA production; it also displays E3-ubiquitin ligase activity *in vitro*. DCAFs act as substrate receptors for E3 ligases and may couple ubiquitination with histone methylation. Here, structural alignment and mutation of signature WDxR motifs in Raf1 indicate that it is a DCAF for CLRC. We demonstrate that Raf1 promotes H3K9 methylation and siRNA amplification via two distinct, separable functions. The association of the DCAF Raf1 with Cul4-Rik1 is critical for H3K9 methylation, but dispensable for processing of centromeric transcripts into siRNAs. Thus the association of a DCAF, Raf1, with its adaptor, Rik1, is required for histone methylation and to allow RNAi to signal to chromatin.

## Author Summary

Heterochromatin is a specialized form of chromatin which is frequently assembled on DNA sequences with little or no coding potential. Heterochromatin formation involves specific post-translational modifications of histone tails (e.g. methylation of histone H3 on lysine 9). In fission yeast, *Schizosaccharomyces pombe*, heterochromatin is found at centromeres, telomeres, and the mating type locus. Heterochromatin integrity at centromeres is important for normal chromosome segregation. The heterochromatin associated repeats at fission yeast centromeres are known to be transcribed, and these non-coding transcripts are processed into siRNAs. siRNA production is required for establishment and maintenance of H3K9 methylation. But H3K9 methylation itself is required for siRNA production. It is not known how these two processes are coupled. In this study we use structural modelling and genetic analyses to demonstrate that the heterochromatin component Raf1 plays an essential role in coupling H3K9 methylation and siRNA production. Our analyses show that the heterochromatin factors Rik1 and Raf1 can be structurally aligned with Cul4-E3 ubiquitin ligase components DDB1 and DDB2, respectively. We show that specific mutations impair the association of Raf1 with Rik1 and prevent H3K9 methylation but not siRNA production. These functional studies provide mechanistic insights into how siRNA production and chromatin modification are integrated.

## Introduction

Silencing mechanisms mediated by small RNAs occur in most eukaryotes. RNA interference (RNAi) can reduce gene expression post-transcriptionally by cleaving homologous transcripts or by inhibiting their translation [1]. Small RNAs can also act in the nucleus, inducing transcriptional gene silencing (TGS) [2], [3]. Although siRNA-directed modification of homologous chromatin is widespread in eukaryotes, the details of how siRNAs mediate such events remain limited in most systems. [4], [5]. In plants and fungi, the link between siRNA-directed DNA/chromatin modification and heterochromatin formation is well established [2], [3]. The fission yeast, *Schizosaccharomyces pombe*, is a powerful system in which to study RNAi-directed heterochromatin formation in part because it contains single non-essential genes encoding each of the key RNAi components.

In fission yeast, siRNAs are important for heterochromatin formation on the centromeric outer repeats (composed of *dg* and *dh* elements) and other chromosomal locations. Although marker genes inserted at these loci are silenced [6], centromeric repeats are bi-directionally transcribed by RNAPII, producing double-stranded RNA (dsRNA) [2], [3], [7], [8], [9]. Dicer (Dcr1) cleaves these non-coding transcripts into siRNAs that guide the Argonaute (Ago1)-containing RITS complex to homologous nascent transcripts by sequence complementarity. Chromatin-bound-RITS recruits the histone methyltranferase Clr4^SuVar3-9^ (a CLRC component; see below) to centromeric repeats via the linker protein Stc1 [10]. Clr4 methylates lysine 9 of histone H3 (H3K9me), providing binding sites for the chromodomain proteins Swi6, Chp1 (a RITS component) and Chp2 [11], [12], [13], [14]. Two different non-mutually exclusive models have been proposed to explain how heterochromatin, once assembled, silences centromere repeat transcription. In the ‘transcriptional gene silencing’ (TGS) model, heterochromatin factors directly repress transcription [15]. In the ‘co-transcriptional gene silencing’ (CTGS) model the repeats are continuously transcribed and silencing is due to the efficient processing of transcripts to siRNA [16]. In wild-type cells, siRNA production and H3K9 methylation are coupled processes: deletion of genes encoding CLRC components results not only in loss of H3K9 methylation but also in loss of siRNA [17], [18], [19]. Similarly, deletion of genes encoding RNAi components abrogates siRNA production and reduces H3K9me [9]. However, cells expressing only mutant histone H3 (H3K9R) produce some detectable siRNAs, even though H3K9 can not be methylated [20], [21]. Moreover, tethering the Rik1 CLRC component to *ura4* RNA triggers silencing of the *ura4^+^* gene independently of other CLRC subunits, but requires RNAi [21]. Thus CLRC itself, rather than its substrate H3K9, promotes siRNA production independently of H3K9. However, it is not known how CLRC integrates histone methylation with siRNA generation.

CLRC is composed of the histone methyltransferase Clr4, the β-propeller protein Rik1, the cullin protein Cul4, the WD-40 protein Raf1 (Clr8/Cmc1/Dos1) and Raf2 (Clr7/Cmc2/Dos2), which contains a RFTS domain [17], [22], [23], [24], [25]. Although it is not known whether CLRC acts as an E3-ubiquitin ligase *in vivo*, CLRC purified from cells exhibits E3 ligase activity *in vitro* [24]. E3 ligases regulate a variety of biological processes by bridging the E2 ubiquitin conjugating enzyme to specific substrates, allowing their ubiquitination (for review see [26]). Histone methylation is frequently regulated by ubiquitination but the mechanistic details remain unknown [27].

The Cullin-RING ligases (CRLs) are the largest family of multi-subunit E3 ligases in eukaryotes and are formed by a neddylated member of the cullin family scaffold (CUL1-CUL5), a RING finger protein (RBX1 or RBX2) and an adaptor protein that bridges the CUL/RING scaffold to substrate receptors [28]. Amongst CRLs, CRL4 is known to ubiquitinate histones [29], [30]. In the CRL4 complex, DDB1 acts as the adaptor. Affinity purification of DDB1 from human cells identified various WD-40 proteins as possible substrate receptors termed DCAFs (DDB1 and CUL4 Associated Factors) [31], [32], [33], [34]. Several DCAFs (WDR5, RBP5 and EED) not only interact with DDB1 but are also members of histone methyltransferase complexes [31], [32], [33], [34]. Although it is not known whether the interaction between these DCAFs and CUL4/DDB1 leads to ubiquitination of substrates, it might have important functional consequences since the knock-down of CUL4 or DDB1 reduces histone methylation [31]. Many DCAFs contain a specific WDxR motif that is important for their association with DDB1 [31], [32], [33], [34].

In *S. pombe*, Cul4 associates with the canonical adaptor Ddb1 [35] and with Rik1 (which shares similarity with Ddb1; [36]) in CLRC [17], [22], [23], [24]. Both Cul4-Ddb1 complex and CLRC affect heterochromatin integrity and neddylation of Cul4 is required for H3K9 methylation of heterochromatic regions [22], [37]. This suggests that E3 ligase activity is involved in heterochromatin formation. However, heterochromatin defects observed in neddylation-defective Cul4 (*cul4-K680R*) could arise either from impaired Cul4-Ddb1 activity or from defective CLRC function. Furthermore, although Rik1 exhibits some similarity to Ddb1 and thus might act as an adaptor, recent analyses suggest that Rik1 is primarily an RNA binding protein which associates with centromeric transcripts via its CPSF-A domain [21]. Thus, it remains to be determined how CLRC contributes to heterochromatin integrity and whether CLRC acts as a DDB1-related E3 ligase which promotes heterochromatin formation *in vivo*.

Here we demonstrate that the CLRC components Rik1 and Raf1 can be structurally aligned with the human adaptor DDB1 and its DCAF DDB2, respectively. Our analyses are consistent with Raf1 acting as a DCAF for CLRC that contributes to siRNA amplification and H3K9 methylation by two distinct and separable routes. Our findings provide mechanistic insights into how siRNA production is integrated with chromatin modification via CLRC.

## Results

### Raf1 is a chromatin associated component of the CLRC complex

**Figure 1:**
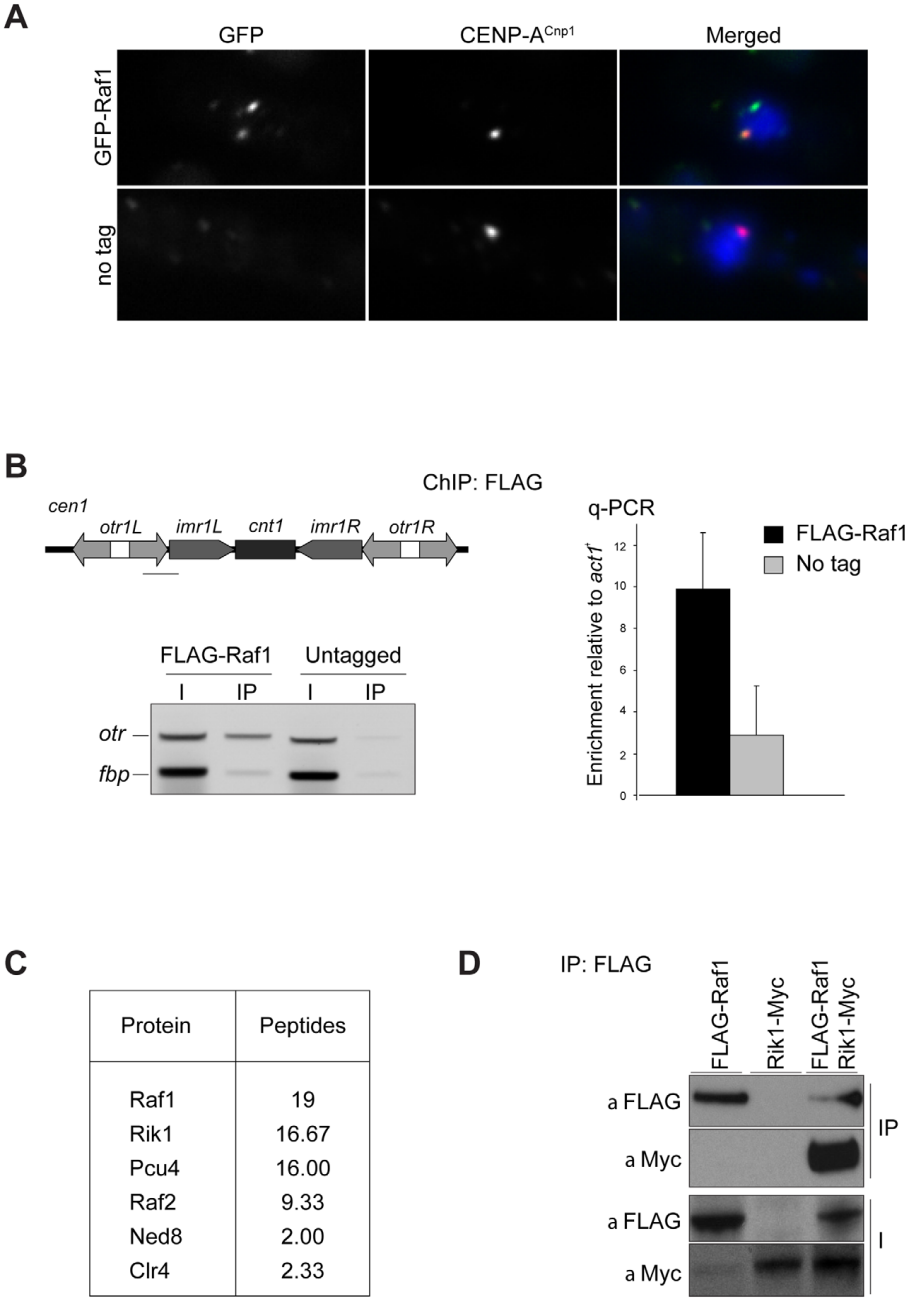
Raf1 is a chromatin-associated CLRC component. (A) Immunolocalisation of GFP-Raf1 (top panel) or untagged control (bottom panel) localisation. Representative images of fixed cells: GFP-Raf1 (green), centromere specific protein CENP-A^Cnp1^ (red), DAPI stained DNA (blue). (B) FLAG-Raf1 ChIP. Diagram shows position of *cen1* primers used (black bar). FLAG-Raf1 or untagged controls cells were analysed by multiplex PCR (*otr* enrichment relative to *fbp1^+^* control - left) or by qPCR (*otr* enrichment relative to *act1* ^+^ - right). I: input; IP: immunoprecipitation. (C) Proteins reproducibly detected in FLAG-Raf1 IPs by LC-MS/MS. Average peptide numbers identified in replicas is shown. (D) Raf1-FLAG IP analysed with anti-FLAG or with anti-Myc to detect Rik1-Myc.

Components involved in heterochromatin formation are typically localised in distinct chromatin-associated foci [14], [38]. In contrast, previous studies overexpressing GFP-Raf1 from a strong promoter showed that it is a nuclear protein with no obvious chromatin localisation [23], however, this might not reflect its true subcellular localisation. Indeed, the genome-wide distribution of Raf1 suggests that it interacts predominantly with heterochromatic loci [13]. To reassess Raf1 localisation, we examined cells expressing GFP-Raf1 from its native promoter and found that it decorates several distinct chromatin-associated foci, the largest of which lies adjacent to the clustered kinetochores (Figure 1A). ChIP confirmed that FLAG-Raf1 associates with centromeric repeats (Figure 1B). We conclude that Raf1 is a chromatin-associated protein concentrated at heterochromatin.

Biochemical purification of CLRC components Rik1, Raf2 and Clr4 identified Raf1 as an interacting partner [17], [22], [24]. In contrast, two-step purification of TAP-Raf1 identified the histone demethylase Lid2 plus Cul4 and Rik1, but apparently not Raf2 and Clr4, known CLRC subunits [39]. This raises the possibility that Raf1 is a component of two distinct complexes: CLRC (Cul4/Rik1/Raf1/Raf2/Clr4) and an alternative Lid2/Cul4/Rik1/Raf1 complex. To further investigate this, we affinity selected FLAG-Raf1 from cell lysates and identified associated proteins by mass spectrometry. Silencing assays indicate that FLAG-tagged Raf1 is functional (Figure S1A). Single step FLAG affinity purification is less stringent than the two-step TAP affinity purification and was performed in mild conditions to identify as many Raf1 interacting proteins as possible. Although we detect all CLRC components, Lid2 peptides were absent (Figure 1C). Moreover, while Rik1 coimmunoprecipitated with Raf1 (co-IP; Figure 1D), Rik1 could not be detected in Lid2-TAP IPs (Figure S1B). We conclude that Raf1 mainly associates with the known CLRC components.

### Raf1 is a DDB2-like WDxR protein

**Figure 2:**
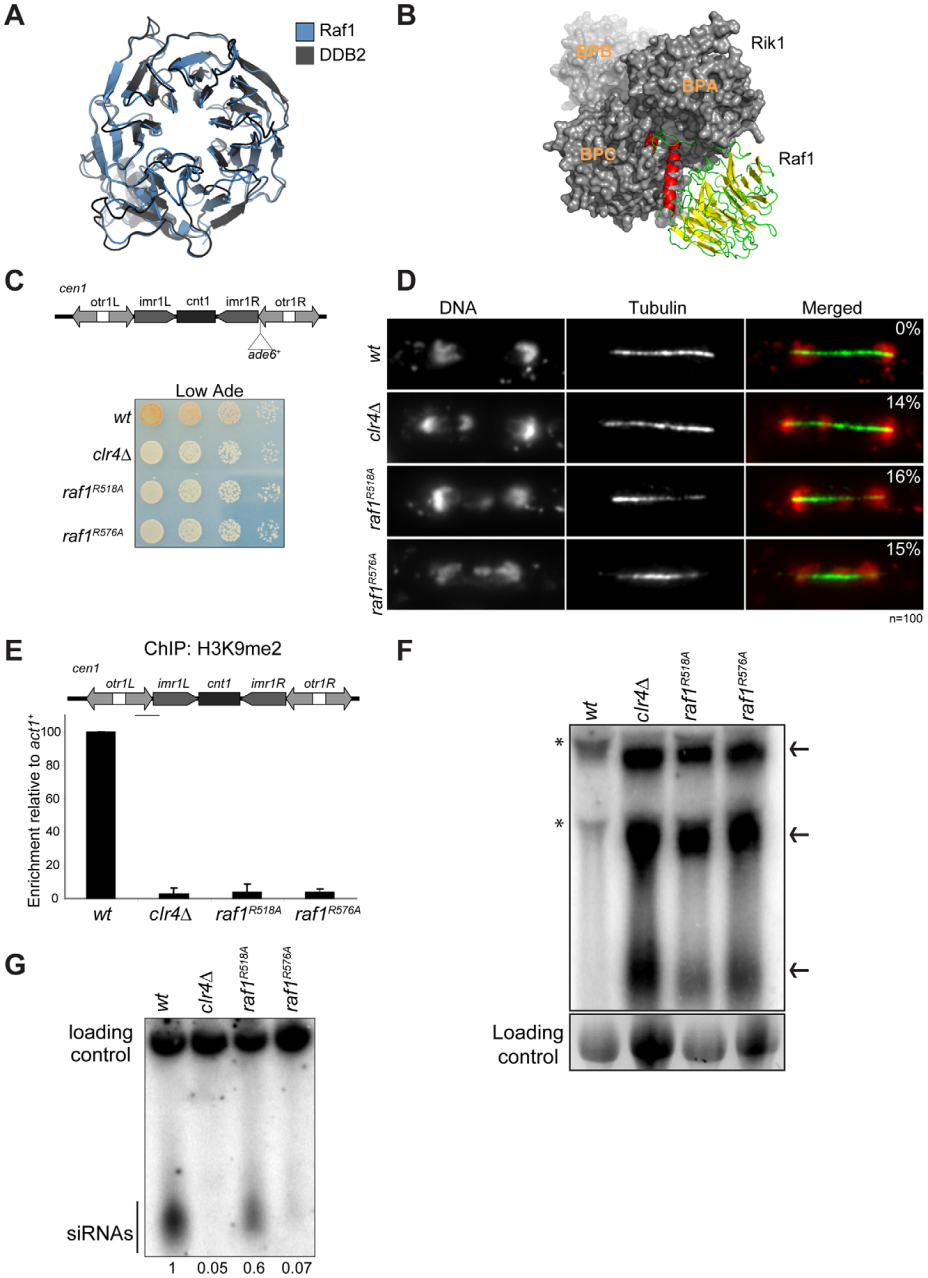
Raf1 is the DCAF for CLRC with WDxR motifs essential for heterochromatin formation. (A) Structural alignment of Raf1 (blue) with human DCAF DDB2 (grey). (B) Structural model of Rik1 (based on DDB1: grey) associated with Raf1 (red, green and yellow). In Raf1, the N-terminal helix (red) and the β-propeller (green and yellow) make specific interaction with Rik1. (C) Centromere silencing assay. Position of *ade6* ^+^ marker gene in *cen1*. Wild-type cells with silenced *cen1:ade6* ^+^ form red colonies; loss of silencing causes white colonies. (D) Lagging chromosomes in anaphase. Representative images of fixed cells stained with DAPI (red) and anti-tubulin (green) and % anaphase cells with lagging chromosomes. (E) H3K9me2 ChIP; levels associated with *cen-dg* relative to *act1* ^+^, normalised to wild-type. Error bars: standard deviation (SD). (F) Northern: unprocessed *otr* transcripts (arrows) in wt, *clr4Δ*, *raf1-R518A* and *raf1-R576A* cells. Loading control: rRNA. (*): rRNA background. (G) Northern: centromeric siRNAs in wt, *clr4Δ*, *raf1-R518A* and *raf1-R576A* cells. Loading control: snoRNA58.

In CRL4 complexes DDB1 is the adaptor that recruits WD-40-containing DCAF proteins [28]. In CLRC, Rik1 is a DDB1-related protein that interacts with Raf1 [23]. Thus, Rik1 and Raf1 might represent the adaptor and the DCAF, respectively, for CLRC. To test these possibilities, we performed structural alignments of Rik1 and Raf1 and found that they can be modelled on DDB1 and on the DCAF DDB2 (Rik1: DOPE score −11385; Raf1: GA431 score 0.6 respectively) (Figure 2A and 2B). This model reveals that, like human DDB1, Rik1 is composed of three WD-40 β-propeller domains (BPA, BPB and BPC) composed of 21× WD-40 repeats and a C-terminal helical domain (Figure 2B and S2A). Raf1, like DDB2, is predicted to contain an N-terminal helical domain and a 7-bladed WD-40 β-propeller domain forming a ring (Figure 2A and 2B). Unlike DDB2, the helical and WD-40 domains of Raf1 are separated by an additional domain of unknown function (Figure S2B). As in the DDB1/DDB2 complex, our model predicts that the Raf1 helical domain can be inserted into the Rik1 BPA-BPC cleft and the ‘top’ of the β-propeller ring interacts with the ‘bottom’ surface of Rik1 (Figure 2B).

Alignment of the Raf1 WD-40 repeats with those of other known DCAFs revealed that Raf1 contains two WDxR motifs (1: aa515–518 and 2: aa573–576) (Figure S3A). WDxR motifs represent a ‘DCAF signature’ and are important for docking DCAFs to DDB1 [31], [32], [33], [34]. Our Raf1 model indicates that these two WDxR motifs are located on the surface of the β-propeller ring and might provide specific sites for interactions with other factors (Figure S3E). To test the importance of Raf1 WDxR motifs, we mutated the endogenous *raf1* gene in *S. pombe* to express FLAG-Raf1-R518A or FLAG-Raf1-R576A. Western analysis indicated that both mutant proteins are expressed at levels similar to wild-type FLAG-Raf1 (Figure S3B). All components of CLRC are required for heterochromatin integrity and consequently for the transcriptional silencing of marker genes placed within centromeric heterochromatin, and centromere function [6]. Like *clr4Δ* cells, heterochromatin-mediated silencing of *cen1:ade6^+^* is disrupted in *raf1-R518A* and *raf1-R576A* mutants, as indicated by white/expressing rather than red/silent colonies (Figure 2C). Moreover, both mutations impair heterochromatin dependent silencing at the mating type locus (Figure S3D).

Centromeric heterochromatin mediates robust sister-centromere cohesion and is therefore required for accurate chromosome segregation during mitosis [40], [41]. Defective heterochromatin causes a quantifiable increase in the frequency of lagging chromosomes on late anaphase spindles [42]. Cells bearing the *raf1-R518A* or *raf1-R576A* mutation exhibit a frequency of lagging chromosomes equivalent to *clr4Δ* cells, indicating that this centromeric function of heterochromatin is disrupted (Figure 2D).

Heterochromatin formation requires processing of centromeric transcripts into siRNA and methylation of H3K9 (H3K9me). Deletion of any gene encoding a CLRC component results in loss of H3K9me, accumulation of centromeric transcripts and a dramatic reduction of siRNA levels [17], [18], [19]. ChIP analyses indicate that H3K9me2 is reduced to background levels in *raf1-R518A* and *raf1-R576A* cells (Figure 2E). Importantly, Clr4 levels are equivalent to wild-type in both mutants (Figure S3C). Consistent with this loss of H3K9me2 and as observed in *clr4Δ* cells, high levels of centromeric transcripts also accumulate (Figure 2F). This is due to increased transcription given that higher levels of RNAPII are detected on centromeric repeats in both mutants compared to wild-type cells (Figure S5E). However, *raf1-R576A* cells contain low levels of centromeric siRNAs whereas *raf1-R518A* cells retain high levels of these siRNAs (Figure 2G and S3F). Our structural model predicts that R518 resides on the surface of Raf1 that interacts with Rik1 and predicts that the R518A mutation may specifically impair Raf1-Rik1 interactions (Figure S3G). In contrast, R576 is more distantly located from Rik1 and may have more profound structural effects.

Together our findings demonstrate that the CLRC complex is likely to adopt a canonical Cul4-E3 ligase architecture and that Raf1 is a DCAF for CLRC. Moreover, we show that WDxR motifs are required for heterochromatin integrity and that the *raf1-R518A* mutation separates the function of CLRC in methylating H3K9 from its role in generating siRNAs.

### The *raf1-1* mutation conditionally disrupts heterochromatin without affecting siRNA generation

**Figure 3:**
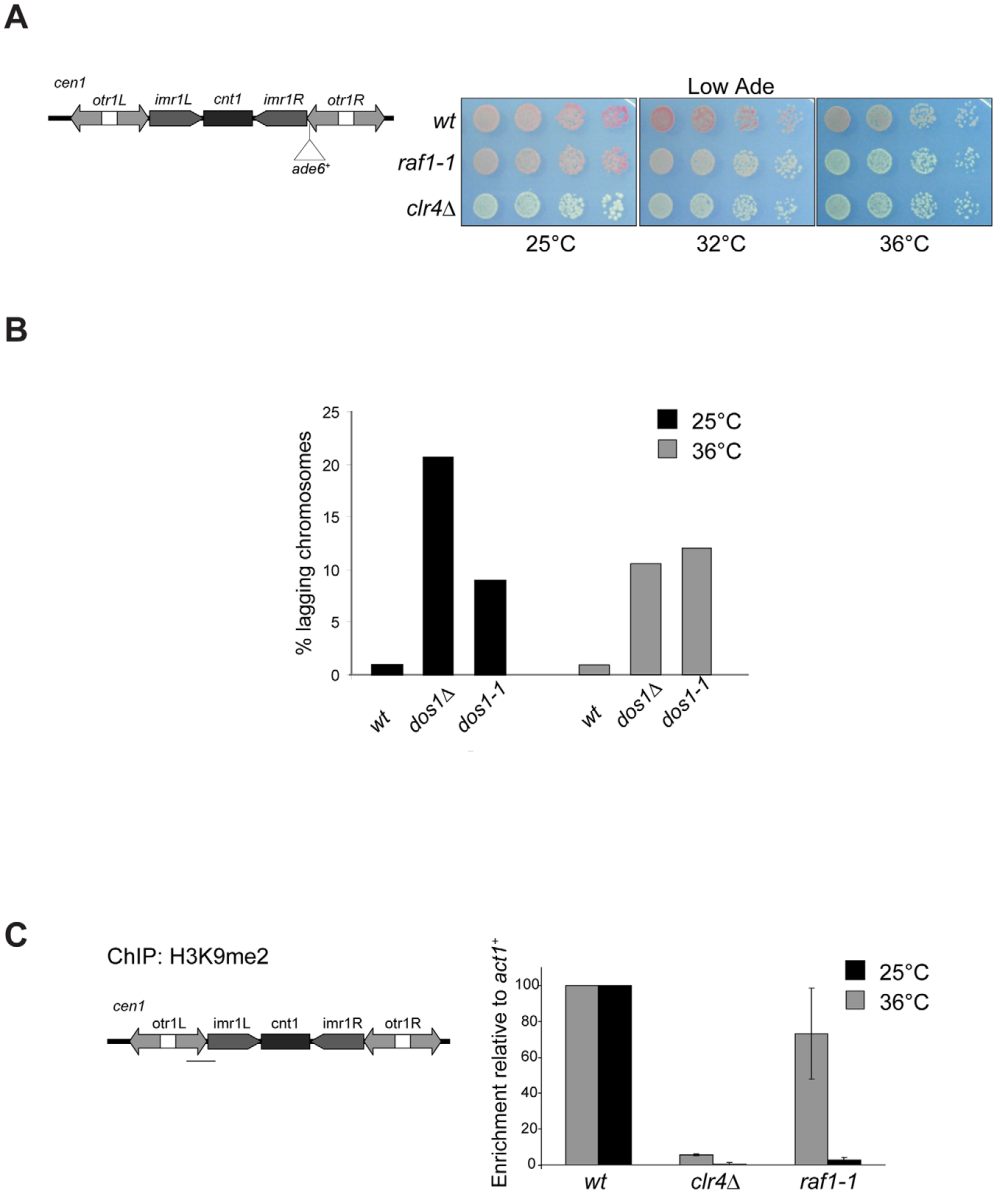
*raf1-1* conditionally disrupts heterochromatin. (A) *cen1:ade6* ^+^ silencing assay in wt, *clr4Δ* or *raf1-1* cells at 25°, 32° or 36°C. (B) Percentage of lagging chromosomes in anaphase of wt, *raf1Δ* and *raf1-1* cells at 25°C or 36°C. Cell lacking heterochromatin display higher rates of chromosome missegregation at lower temperature [42]. (C) H3K9me2 ChIP: levels associated with *cen-dg* relative to *act1* ^+^ in *clr4Δ* and *raf1-1* cells normalised to wild-type at 25° or 36°C. Error bars: SD.

**Figure 4:**
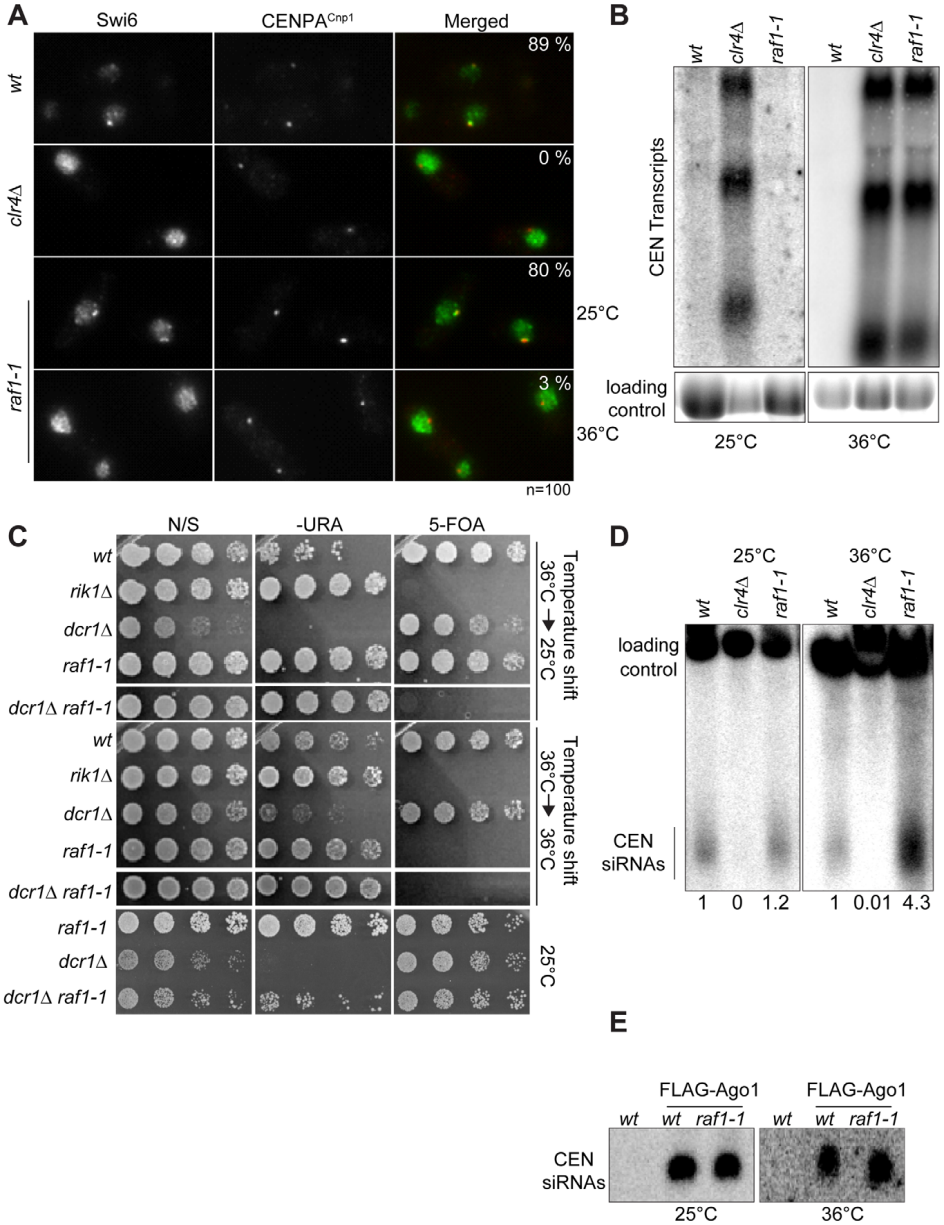
Heterochromatin, but not siRNA production, is erased in *raf1-1* cells. (A) Swi6 localisation in indicated cells at 25° or 36°C. Representative images of fixed cells with Swi6 (green) and CENP-A^Cnp1^(red). % cells with Swi6 localised at centromeres. (B) Northern: unprocessed *otr* transcripts in indicated cells at 25° or 36°C. Loading control: rRNA. (C) *mat3-M:ura4* ^+^ silencing assay of cells grown at 36°C and shifted to 25°C (top panel) or kept at 36°C (bottom panel). non-selective (N/S), –URA or 5-FOA plates indicated. Silencing (wt, *dcr1Δ* and *raf1-1* at 25°C) allows little growth on -URA but good growth on 5-FOA. Loss of silencing (*rik1Δ*, *raf1-1* at 36°C and *raf1-1 dcr1Δ* at 25° or 36°C) results in good growth on –URA and 5-FOA sensitivity. (D) Northern: centromeric siRNA in indicated cells at 25° or 36°C. Loading control: snoRNA58. (E) Northern: FLAG-Ago1-associated siRNA from indicated cells at 25° or 36°C.

To dissect mechanisms governing heterochromatin assembly and maintenance, we screened for temperature-sensitive mutants that disrupt heterochromatin at the restrictive temperature (36°C) but not the permissive temperature (25°C) and isolated the *raf1-1* mutation. *raf1-1* produces a protein with a missense mutation (T495I) in the third WD-40 repeat; this residue is conserved in fungi suggesting that it is important for Raf1 function (Figure S3A). Similar levels of mutant FLAG-Raf1-1 and wild-type FLAG-Raf1 proteins are detected at 36°C thus phenotypes are not due to Raf1-1 degradation (Figure S4A). Marker gene silencing within centromeric repeats (*cen1:ade6^+^* or *cen1:ura4^+^*), the mating type locus (*mat3-M:ura4^+^*) and at telomeres (*tel1L:his3^+^*) is alleviated in *raf1-1* cells at 36°C, but not 25°C (Figure 3A and Figure S4B, S4C). Consistent with defective centromeric heterochromatin integrity, a high frequency of lagging chromosomes is observed in late anaphase *raf1-1* cells at 36°C (Figure 3B). Moreover, H3K9me2 levels on centromeric repeats in *raf1-1* cells are similar to wild-type cells at 25°C but negligible at 36°C (Figure 3C). Consistent with reduced H3K9me2, Swi6 localisation and unprocessed centromere repeat transcript accumulation is temperature dependent in *raf1-1* cells (Figure 4A and 4B). Importantly, Clr4 levels are unaffected by the *raf1-1* mutation (Figure S4B).

Although we detect no H3K9me2 on centromere repeats in *raf1-1* at 36°C, it is possible that residual levels of H3K9me2 remain. To determine if heterochromatin is erased at 36°C, a heterochromatin establishment assay was performed. At the mating type locus, active RNAi is required to establish heterochromatin, but once assembled, RNAi is not required to maintain heterochromatin [43]. Hence, loss of *dcr1*/RNAi has no impact on mating type locus silencing (i.e. *mat3-M:ura4^+^*). However, when presented with a naive template, *dcr1Δ* cells fail to assemble heterochromatin *de novo* and are unable to silence *mat3-M:ura4^+^*. To test whether the *raf1-1* mutant erases heterochromatin structures, a *raf1-1 dcr1Δ* double mutant was generated at 36°C and *mat3-M:ura4^+^* silencing assessed after shifting cells to 25°C. Silencing of *mat3-M:ura4^+^* could not be established in this double mutant following a shift down from 36° to 25°C, but remained intact in the *raf1-1 dcr1Δ* double mutant generated at 25°C (Figure 4C). This demonstrates that *raf1-1* cells are unable to form heterochromatin without RNAi and indicates that heterochromatin is completely erased by the *raf1-1* mutation at 36°C.

Surprisingly, unlike *clr4*Δ and *raf1*Δ mutants, centromeric siRNAs are produced at wild-type levels in *raf1-1* cells at both temperatures (Figure 4D, Figure S4E and S5D). It is possible that siRNA levels remain high because *raf1-1* inhibits the degradation of pre-existing siRNAs. However, high siRNA levels remain in *raf1-1* cells which have undergone 144 divisions at 36°C; pre-existing siRNA would be diluted out (Figure S4E). Thus, the continual synthesis of siRNAs from centromere repeat transcripts must be unaffected by the defect in *raf1-1*. Moreover, these siRNAs are loaded into Ago1/RITS as indicated by their association with FLAG-Ago1 (Figure 4E). We conclude that as with *raf1-R518A*, the *raf1-1* mutation uncouples siRNA production from H3K9 methylation.

### Methylation of H3K9, but not siRNA production, depends on an intact CLRC complex

**Figure 5:**
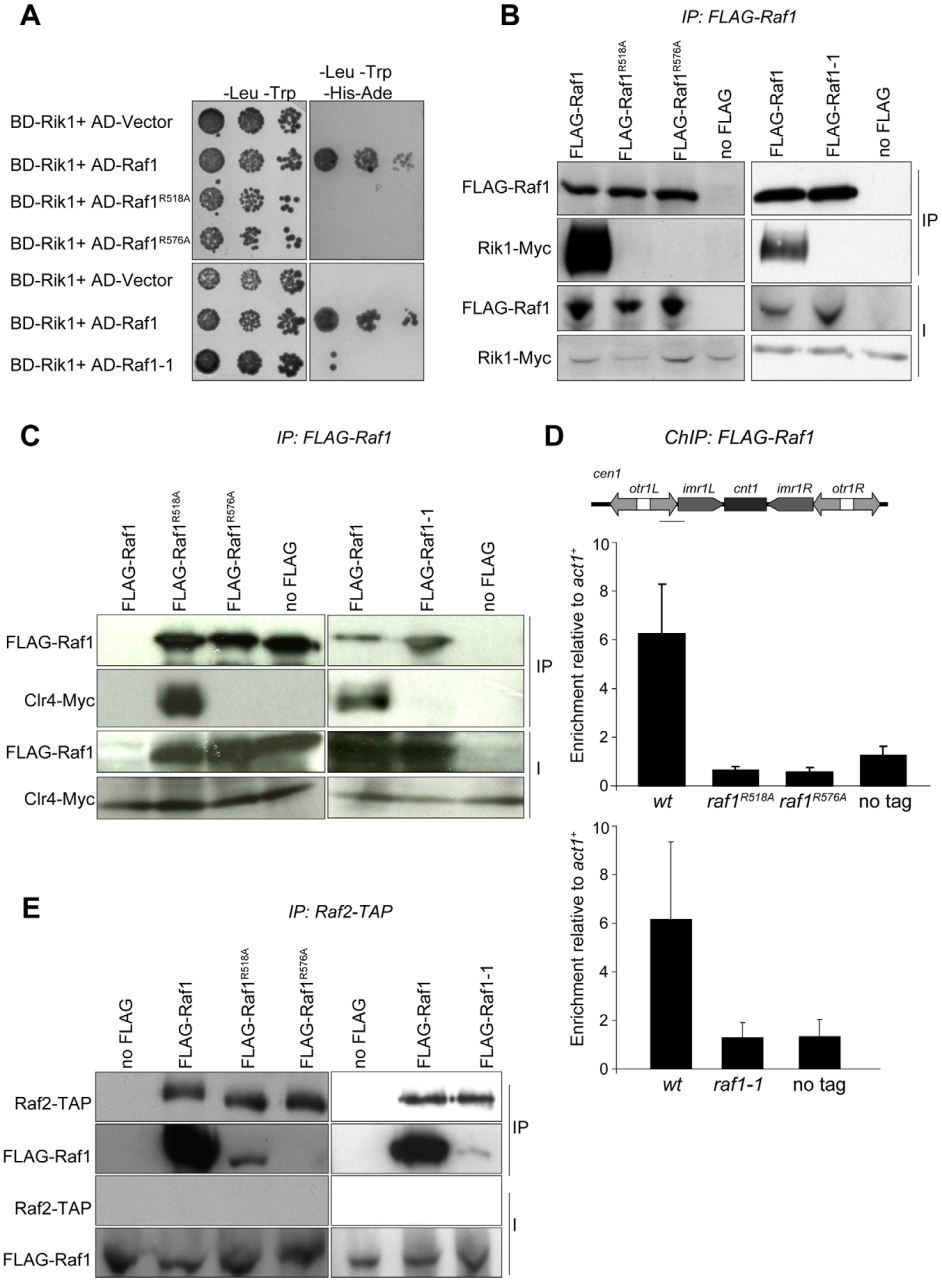
Raf1 mutations disrupt interaction with Rik1. (A) Yeast two-hybrid assay. Interaction of Raf1 with Rik1 is indicated by growth on -Leu, -Trp, -His, -Ade plates. BD and AD: GAL4 *B*inding or *A*ctivation *D*omain fusions, respectively. (B) Western of FLAG-Raf1, FLAG-Raf1-R518A FLAG-Raf1-R576A and FLAG-Raf1-1 IPs analysed for FLAG-Raf1 and Rik1-Myc (right: cells grown at 36°C). (C) Western of FLAG-Raf1, FLAG-Raf1-R518A FLAG-Raf1-R576A and FLAG-Raf1-1 IPs analysed for FLAG-Raf1 and Clr4-Myc (right: cells grown at 36°C). (D) FLAG-Raf1, FLAG-Raf1-R518A, FLAG-Raf1-R576A and FLAG-Raf1-1 ChIP. Diagram shows *cen1* primers used (black bar). Enrichment of FLAG-tagged proteins or untagged control were analysed by qPCR of *otr* relative to *act1* ^+^(bottom: cells grown at 36°C). Error bar SD. (E) Westerns of Raf2-TAP IP analysed for FLAG-Raf1, FLAG-Raf1-1, FLAG-Raf1-R518A and FLAG-Raf1-R576A (right: cells grown at 36°C).

The interaction between the adaptor DDB1 and its DCAFs is known to require intact WDxR motifs in the DCAF [32], [33], [34]. Our structural alignment predicts that the *raf1-1* T495I mutation is located on the ‘top’ surface of the WD-40 ring that interacts with Rik1 (Figure S5A). It is therefore possible that our specific *raf1* mutations impair the ability of Raf1 to associate with Rik1 and that this interaction is essential for establishing and maintaining H3K9 methylation on heterochromatic loci. Indeed two-hybrid assays reveal that the binding of Raf1 to Rik1 [23] is disrupted in both WDxR motif mutants and the *raf1-1* mutation (Figure 5A). All three mutations also impair co-immunoprecipitation of Rik1 with Raf1 from *S. pombe* extracts (Figure 5B). In addition, the association of the Clr4 H3K9 methyltransferase with Raf1 and Rik1 is lost in raf1-*R518A*, *raf1-R576A* and *raf1-1* mutants (Figure 5C and Figure S5B). Recently we identified Stc1 as protein that links the RNAi machinery to CLRC [10]. Consistent with this, these same three specific *raf1* mutations abolish the association of the Rik1 CLRC component with Stc1 (Figure S5C). Furthermore, ChIP analyses indicate that FLAG-Raf1-R518A, FLAG-Raf1-R576A and FLAG-Raf1-1 do not associate with centromeric repeats (Figure 5D). We conclude that binding of Raf1 to Rik1 is required to allow Raf1 to associate with centromeric repeats. This interaction is required to assemble an active CLRC complex in order to establish and maintain H3K9 methylation and hence functional heterochromatin.

The coupling of H3K9 methylation and siRNA production could be achieved by bringing the distinct activities required for both processes together in the same complex. Since the *raf1*Δ and *raf1-R576A* mutations results in loss of siRNAs, Raf1 is clearly required for siRNA production (Figure S5D and Figure 2G). However, the *raf1-R518A* and *raf1-1* mutants lose H3K9 methylation without affecting siRNA synthesis (Figure 2E, 2G; Figure 3C; Figure 4D; Figures S4E and S5D). This suggests that specific interactions between Raf1 and individual proteins in CLRC and/or other unknown factors are sufficient to allow siRNA generation in the absence of H3K9 methylation. An intact CLRC complex may not be required. Indeed, co-immunoprecipitation experiments show that a weak but detectable interaction between Raf1 and Raf2 remains in *raf1-1* and *raf1-R518A*, but not *raf1-R576A* cells (Figure 5E). We conclude that although an intact CLRC complex is dispensable for siRNA synthesis, a minimal Raf1-Raf2 interaction might be sufficient to allow the processing of centromere repeat transcripts to siRNAs.

## Discussion

### The interaction between the E3 ligase adaptor Rik1 and the DCAF Raf1 correlates with methylation of lysine 9 on histone H3

The CLRC complex, harbouring the histone methyltransferase Clr4^Suvar3-9^, performs an essential role in the establishment and maintenance of heterochromatic structures. In addition to Clr4, the complex contains the components Cul4, Rik1, Raf1 and Raf2.

**Figure 6:**
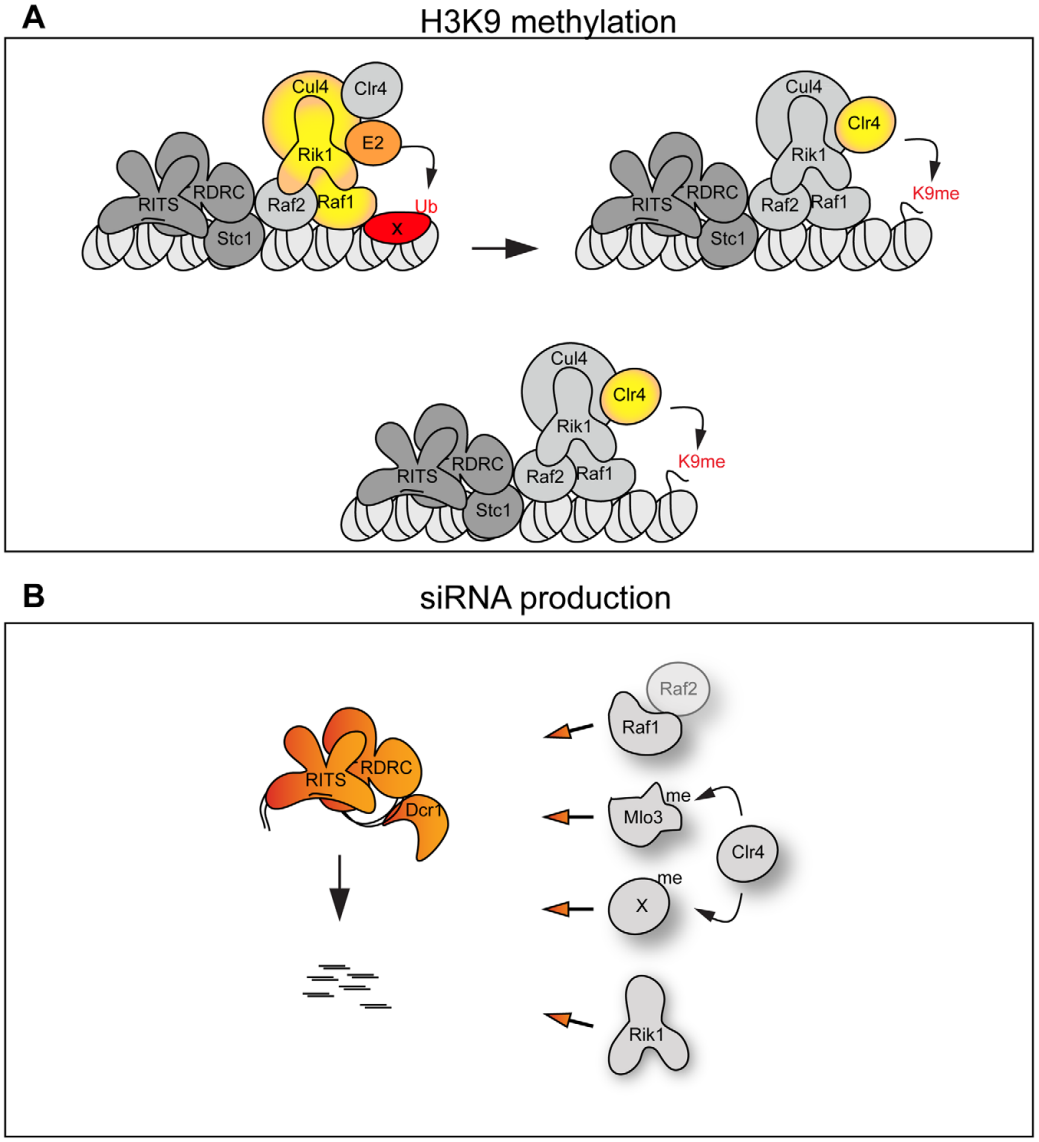
The CLRC complex couples H3K9me and siRNA production. (A) Top panel: The CLRC complex is an active E3 ligase. The DCAF Raf1 binds a specific substrate (X) allowing its ubiquitination. This is essential for methylation of H3K9. Bottom panel: The CLRC complex is not an active E3 ligase. Cul4, Rik1, Raf1 and Raf2 form a protein scaffold allowing the correct targeting of the histone methyltransferase Clr4. (B) Individual CLRC components stimulate processing of centromeric transcripts into siRNAs independently of CLRC complex integrity. Raf1-Raf2, methylation of Mlo3 or other substrates and Rik1 (left) are required for processing centromeric transcripts into siRNAs.

Rik1 is a WD-40 repeat protein that shares homology with the E3 ligase adaptor DDB1 [36]. DDB1 contains 21 WD-40 repeats that form the seven blades of three β-propellers that mediate association with Cul4 and DCAFs. This supports the possibility that Cul4-Rik1 form the core of an E3 ligase [24]. However, the C-terminus of Rik1 also shows homology with the β-propeller domain of cleavage and polyadenylation factor CSPF-A a well-known RNA binding protein [18]. Based on this CPSF-A homology, it has been suggested that Rik1 might bind RNA through this domain [21]. Although we cannot exclude that Rik1 is a bifunctional protein, our manual alignment detects 21 WD-40 repeats within Rik1 allowing it to be structurally aligned along its entire length with DDB1 (Figure 2B and Figure S2A). In our model, the CSPF-A homology domain corresponds to the β-propeller BPC involved in the interaction with the DCAF Raf1 (Figure 2B and Figure S2A). Moreover, we also show that Raf1 can be structurally aligned to the DCAF DDB2, the partner of DDB1 (Figure 2A, 2B and Figure S2B). As expected for a *bona fide* DCAF, Raf1 associates directly with the putative substrate adaptor Rik1 and contains two signature WDxR motifs which we show are required for the association of Raf1 with Rik1 and for H3K9 methylation and heterochromatin formation (Figure 2B and Figure S3). This suggests that the CLRC complex can adopt the architecture of a Cul4 E3 ligase in which Rik1 is the adaptor protein bridging the interaction between Cul4 and the DCAF Raf1 (Figure 6A). In addition, we show that the association of the DCAF Raf1 with Rik1 within an intact CLRC complex is critical for H3K9 methylation but it is dispensable for the processing of centromeric transcripts into siRNAs (Figure 2, Figure 4, and Figure 5).

We envisage two alternative scenarios that can explain how the Cul4-Rik1-Raf1 complex might mediate H3K9 methylation (Figure 6A). First, it is possible that the CLRC is an active E3 ligase and that mono- or poly-ubiquitination of specific factors must occur to allow methylation of H3K9. Thus the DCAF Raf1 would be essential for the recognition and ubiquitination of key specific substrate(s) (Figure 6A top panel). In other systems, Cul4-E3 ligases have been shown to ubiquitinate histones [29], [30], it is possible that a specific histone residue needs to be ubiquitinated to allow methylation of H3K9. E3 ligase activity has been shown to associate with affinity selected CLRC *in vitro* [24], however, we have been unable to detect this activity in similar experiments. This suggests that CLRC E3 ligase activity may be particularly inefficient and/or the ubiquitination events that it mediates are transient.

The alternative scenario is that the CLRC complex is not an active E3 ligase *in vivo* and that Cul4, Rik1, Raf1 and Raf2 just form a protein scaffold that acts to target Clr4 methyltransferase to heterochromatic repeats, independently of ubiquitination (Figure 6A bottom panel). In this case the specific mutations which impair the Rik1-Raf1 interaction may just disrupt the scaffold so that Raf1 is not targeted to the heterochromatic repeats and Clr4 no longer associates with Rik1 and Raf1. Interestingly, the DCAF DDB2 has been shown to bind DNA with the ‘bottom’ surface of its WD-40 ring [44]. Similarly, the WD-40 ring of the DCAF WDR5 specifically binds the histone H3 N-terminal tail methylated on lysine 4 [45], [46], [47]. Thus, it is possible that the WD-40 ring of the DCAF Raf1 also binds DNA or histones to allow the correct targeting of the histone methyltransferase Clr4 to heterochromatic repeats.

### Raf1 function in H3K9 methylation and RNAi are separable

CLRC plays a dual role in heterochromatin formation: it harbours the histone methyltransferase Clr4 and hence it is responsible for H3K9 methylation, it also mediates siRNA production since cells lacking any single CLRC subunit have low centromeric siRNA levels (Figure S5D and [17], [18]). Therefore, in wild-type fission yeast H3K9 methylation and siRNAs synthesis are coupled. This ensures formation of heterochromatin at centromeres, telomeres, and the mating type locus, and prevents promiscuous silencing at other chromosomal regions. However, cells expressing only mutant histone H3 (H3K9R) have been shown to produce some detectable siRNAs, even though H3K9 can not be methylated [20], [21]. Such analyses implicate CLRC itself, rather than its known substrate H3K9, in promoting siRNA production independently of H3K9.

Here we have isolated two mutations in the *raf1* gene (*raf1-1* and *raf1R518A*) that destroy CLRC integrity without affecting siRNA levels. Although we cannot exclude that a minimal CLRC complex is still present in these mutants, our results indicate that individual CLRC components can stimulate processing of centromeric transcripts into siRNAs independently of CLRC complex integrity (Figure 6B). It remains to be determined how CLRC components promote this H3K9 methylation-independent siRNA production. Our analyses of specific *raf1* mutants suggests that the putative E3 ligase activity of the CLRC complex is not required for siRNA synthesis given that in *raf1-1* and *raf1-R518A* mutants (predicted to impair the E3 ligase activity of the complex) siRNA levels remain high (Figure 2G and Figure 4D). One possibility is that Clr4 can methylate specific substrates independently of CLRC integrity and that this is a key event in siRNA production [21]. In accordance with this, Clr4 was recently shown to methylate the RNA processing factor Mlo3 and this methylation correlates with high siRNA levels [48]. However, Clr4-mediated methylation of specific substrates cannot be the only event required to trigger siRNA production since the loss of any CLRC component results in dramatic reduction of siRNA levels. We find that high siRNA levels correlate with a low but detectable Raf1-Raf2 interaction. Complete disruption of this Raf1-Raf2 interaction (as observed in *raf1-R576A*) cuts siRNA production to undetectable levels. Interestingly, centromeric repeats are transcribed preferentially in S-phase and Raf2 has been recently shown to interact with Cdc20 (the catalytic subunit of DNA-polymerase ε) [49], [50], [51]. Coordination of DNA replication, siRNA generation and methylation of specific substrates may be essential for heterochromatin establishment and maintenance of heterochromatic structures.

Our analyses suggest that integration of DNA replication, siRNA production and methylation of H3K9 is achieved by bringing the distinct activities required for these processes together in the same protein complex (CLRC). This ensures the assembly of robust heterochromatin structures.

### Cotranscriptional gene silencing plays a subsidiary role in repression of heterochromatic repeats

Two different models have been proposed to explain how heterochromatin could silence centromeric repeats. In the ‘TGS’ model, heterochromatin factors have been proposed to repress transcription at centromeres [15] whereas the ‘CTGS’ model suggests that centromere repeats are continuously transcribed and silencing is caused by the efficient cleavage of centromeric transcripts by RNAi into siRNA [16]. Our analyses demonstrate that the RNAi machinery is active in *raf1-1* cells, so that centromeric transcripts are processed into siRNAs independently of heterochromatin integrity. *raf1-1* cells produce siRNAs at levels similar to wild-type cells and they are loaded into Ago1. However, despite this, high levels of unprocessed centromeric transcripts persist, indicating that RNAi fails to destroy them. The fact that heterochromatin is absent in *raf1-1* and *raf1-R518A* cells and transcripts remain high, even though high levels of homologous Ago1-associated centromeric siRNAs are present, is more compatible with a TGS model where heterochromatin directly represses RNAPII transcription of centromere repeats.

In agreement with this model, RNAPII ChIP clearly shows an increase of RNAPII occupancy in the *raf1* mutants compared to wild-type cells (Figure S5E). This observation is also consistent with the fact that tethered Clr4 can generate heterochromatin and silence marker genes independently of RNAi [52].

The coupling of non-coding transcription with chromatin modification to establish and maintain distinct epigenetic states is a general mechanism of gene regulation in eukaryotes. Links between Cul4-dependent ubiquitination and histone methylation are continuing to emerge in different systems. Further analyses will determine how these activities are integrated to regulate specific chromatin states.

## Materials and Methods

### Strain and plasmid construction

Standard procedures were used for bacterial and fission yeast growth and genetic manipulations [53]. *S. pombe* strains used in this study are described in Table S1. Primer sequences are listed in Table S2.

### Structural modelling

The homology model of Raf1 was created with the program Modeller (9v8) using the structure of DDB2 (PDB code 3EI4) [44]. Iterative rounds of alignment adjustment of the two protein sequences and model building were attempted until the Modeller scores (diagnostic of model quality) were optimised. The homology model of Rik1 was created with the program Modeller (9v8) using 5 templates (PDB code: 2B5L; 317N; 3189; 318C; 3E0C). The generated model (85.2) had the following model scores: RMSD: 1.536; MolPDF: 453.98; DOPE: −11385.

### Chromatin immunoprecipitation

Chromatin immunoprecipitation (ChIP) was performed as described [54] with the following modifications. Cells were fixed in 1% PFA/15 min for H3K9me2 ChIP or in 1% PFA/20 min for RNA Polymerase II ChIP. For ChIP analyses of *raf1-R518A* and *raf1-R576A* strains, cells were grown at 32°C. For ChIP analyses of *raf1-1* strain, cells were kept at the indicated temperature (25°C, 32°C or 36°C) for at least 96 hours. One microliter of monoclonal H3K9me2 antibody (m5.1.1), two microliter of anti-FLAG M2 monoclonal antibody (Sigma, F1804) or five microliters of RNA Polymerase II 8WG16 antibody (COVANCE, MMS-126R) was used per ChIP. Duplex PCR was performed to analyse ChIP samples using oligonucleotides specific to the regions of interest and to the control gene *fbp1* (Table S2). Real-time PCR (qPCR) was performed using the LightCycler 480 SYBR Green I Master (Roche) on a LightCycler 480 Instrument (Roche). qPCR analysis primers are in Table S2. Relative enrichments were calculated as the ratio of product of interest to control product (*act1* ^+^ or *tRNA*) in IP over input. Histograms represent data from three biological replicates analysed in parallel.

### Immunoaffinity purification

Immunoaffinity purifications (IP) for LC-MS/MS analysis were performed as described [55], with the following modifications: 5 g of cells were resuspended in ice-cold-lysis buffer (50 mM Hepes pH7.5, 150 mM KCl, 0.1% NP40). Immunoprecipitation was performed using proteinG Dynabeads resin (Life Technologies) coupled to anti-FLAG M2 antibody (Sigma, F1804) for 15 min. The IP'd material was treated with 500 U Benzonase, washed, subjected to on-bead Tryptic digestion, and prepared for LC-MS/MS analysis as described previously [56].

Co-IPs for Western analysis were performed on 2 g of cells as above but for 1 hr. IP'd material was washed four times with ice-cold lysis buffer (50 mM Hepes pH7.5, 150 mM KCl, 0.1% NP40), resuspended in SDS sample buffer and analysed by SDS-PAGE. For tandem affinity purification (TAP)-tagged strains, Dynabeads coupled to IgG were used (gift from K. Hardwick). For Western analysis the following antibodies were used: anti-FLAG M2 (Sigma, F1804), anti-HA 12CA5 (gift from K. Samejima) and anti-myc (A14) (Santa Cruz sc-789), all at 1∶1000. Further details of IP protocol are provided in Text S1.

### RNA analysis

Northern analysis of long non-coding centromeric transcripts and centromeric siRNAs were performed as described previously [56]. RNA probes are listed in Table S2.

Note: Further details of all experimental procedures are provided in Text S1.

## Supporting Information

Figure S1 Lid2-TAP is not pulled down with F*LA*G-Rik1. (A) Centromere silencing assay. Top: Position of *ade6* ^+^ marker gene in *cen1*. Bottom: Wild-type cells with silenced *cen1:ade6* ^+^ form red colonies. Cells expressing FLAG-Rik1 form red colonies. Loss of silencing results in white colonies, as observed in *raf1Δ* cells. (B) Lid2-TAP IP followed by western with FLAG antibody to detect Rik1-FLAG. I: 5% of input; IP: immunoprecipitation.
(TIF)

Figure S2 Rik1 contains 21 WD-40 repeats and Raf1 can adopt a DDB2-like structure. (A) Detection and comparison of WD-40 repeats in human DDB1 and Rik1. Alignment of DDB1 and Rik1 along with predicted structure. The 21 WD-40 repeats are highlighted in blue which were identified manually in Rik1 by comparison of secondary structures using PSIPRED. (B) Comparison of DDB2 structure (left) with Raf1 model (right). Both proteins contain an N-terminal H-T-H (red) and a 7 bladed β-propeller (green). Unlike DDB2, Raf1 contains an additional domain (blue) of unknown function.
(TIF)

Figure S3 Raf1 contains two WDxR motifs important for protein function. (A) Representative multiple sequence alignment of the C-terminal WD-40 repeats of Raf1 in homologous proteins from fungi. Mutated residues mentioned in the text (T495I, R518A, R576A) are highlighted. (B) Western analyses of untagged, FLAG-Raf1, FLAG-Raf1^R518A^ and FLAG-Raf1^R576A^ from whole cell extracts. Wild-type and mutant Raf1 proteins (arrow) are expressed at similar levels. Asterisk *: indicates cross-reacting band which serves as a loading control. (C) Western analyses of untagged and Clr4-Myc in wild-type, *raf1-R518A* and *raf1-R576A* whole cell extracts. Loading control: Bip1. (D) Assay for silencing at *mat3-M:ura4* ^+^. Plates are non-selective (N/S), lacking uracil (-URA) or supplemented with 5-FOA. Loss of silencing results in growth on -URA and loss of resistance to 5-FOA. (E) Top view of Raf1 β-propeller ring. Residues R518 and R576 are highlighted. (F) Northern: centromeric siRNAs in wild-type, *clr4Δ*, *raf1-R518A* and *raf1-R576A* cells. Loading control: snoRNA58. (G) Rik1-Raf1 model showing the position of residues R518 and R576.
(TIF)

Figure S4 *raf1-1* impairs heterochromatin formation. (A) Western analyses of untagged, FLAG-Raf1 and FLAG-Raf1-1 from whole cell extracts. (B) Western analyses of Clr4-Myc in wild-type and *raf1-1* whole cell extracts. A strain not expressing Clr4-Myc (no Myc) is included as a control. Loading control: Bip1. Cells were grown at 36°C. (C) Assay for silencing at *mat3-M:ura4* ^+^. Plates are non-selective (N/S), lacking uracil (-URA) or supplemented with 5-FOA. Loss of silencing results in growth on -URA and loss of resistance to 5-FOA. (D) Assay for silencing at *tel1L:his3* ^+^. Plates are non-selective (N/S) and lacking histidine (-HIS). Loss of silencing results in growth on –HIS. (E) Lagging chromosomes in anaphase wild-type and *raf1-1* cells at 25°C or 36°C. Representative images of fixed cells with DAPI (red) and anti-tubulin (green). (F) Northern: centromeric siRNAs in wild-type, *clr4Δ*, *raf1-1* cells. Cells were shifted from 25°C to 36°C for the indicated period of time (3, 6 and 12 days resulted in 36, 72 and 144 divisions, respectively, at the restrictive temperature). Loading control: snoRNA58.
(TIF)

Figure S5 *raf1-1*, *raf1-R518A* and *raf1-R576A* disrupts CLRC and heterochromatin integrity. (A) Rik1-Raf1 model showing the position of the T495I mutatedresidue in *raf1-1*. (B) Westerns of Rik1-FLAG IP analysed for Clr4-Myc in wild-type, *raf1-R518A*, *raf1-R576A* and raf1-1 cells. Right: cells grown at 36°C. (C) Westerns of Stc1-FLAG IP analysed for Rik1-Myc in wild-type, *raf1-R518A*, *raf1-R576A* and *raf1-1* cells. Right: cells grown at 36°C. (D) Northern: centromeric siRNAs in wild-type, *raf1-1*, *clr4Δ*, and *raf1Δ* cells. Cells grown at 36°C. Loading control: snoRNA58. (E) RNAPII ChIP in wild-type, *clr4Δ*, *raf1-R518A* and *raf1-R576A* cells. Diagram (left) indicates position of *cen1* primers used (grey bar). RNAPII enrichment (right) was analysed by qPCR relative to tRNA gene primers. Error bar SD.
(TIF)

Table S1 List of strains used in this study.
(DOC)

Table S2 List of primers used in this study.
(DOC)

Text S1 Supplementary experimental procedures.
(DOC)
