# Supplementary figures and images for: Raf1 Is a DCAF for the Rik1 DDB1-Like Protein and Has Separable Roles in siRNA Generation and Chromatin Modification
Source: PLoS Genet. 2012 Feb 2;8(2):e1002499. doi: 10.1371/journal.pgen.1002499 (PMC3271066; doi:10.1371/journal.pgen.1002499)

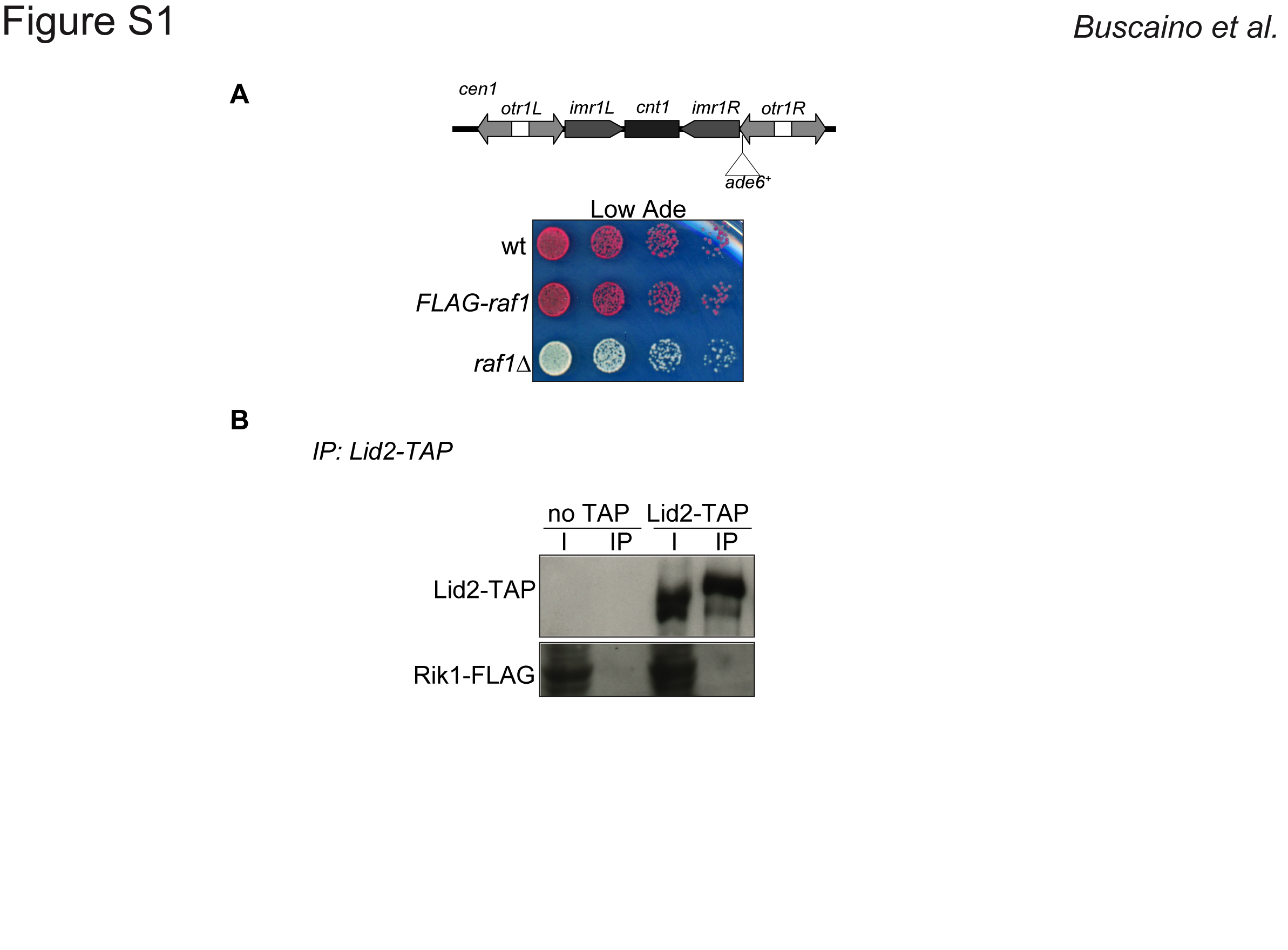

Supplement: Figure S1 — Lid2-TAP is not pulled down with FLAG-Rik1. (A) Centromere silencing assay. Top: Position of ade6 + marker gene in cen1. Bottom: Wild-type cells with silenced cen1:ade6 + form red colonies. Cells expressing FLAG-Rik1 form red colonies. Loss of silencing results in white colonies, as observed in raf1Δ cells. (B) Lid2-TAP IP followed by western with FLAG antibody to detect Rik1-FLAG. I: 5% of input; IP: immunoprecipitation. (TIF) [file pgen.1002499.s001.tif]

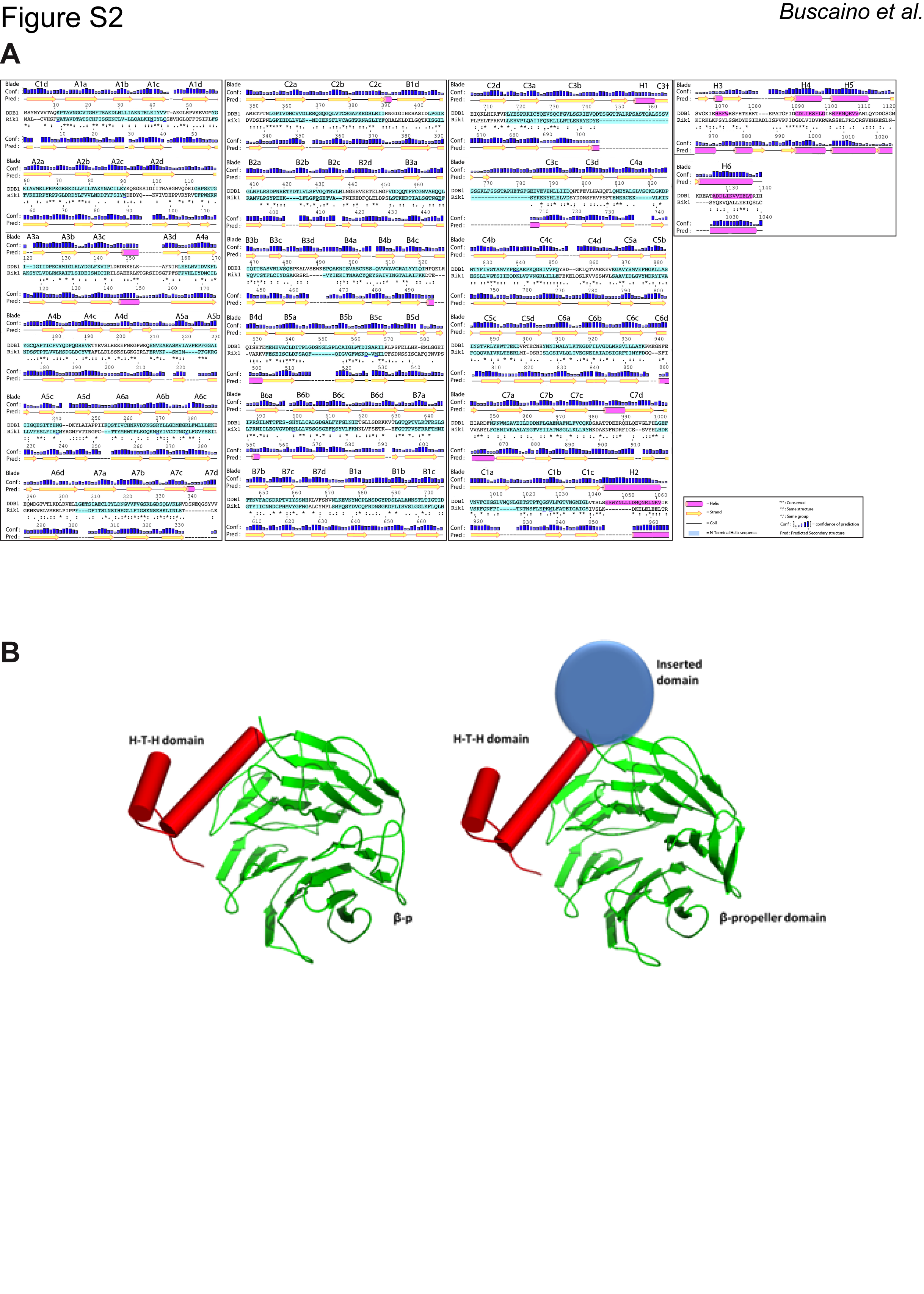

Supplement: Figure S2 — Rik1 contains 21 WD-40 repeats and Raf1 can adopt a DDB2-like structure. (A) Detection and comparison of WD-40 repeats in human DDB1 and Rik1. Alignment of DDB1 and Rik1 along with predicted structure. The 21 WD-40 repeats are highlighted in blue which were identified manually in Rik1 by comparison of secondary structures using PSIPRED. (B) Comparison of DDB2 structure (left) with Raf1 model (right). Both proteins contain an N-terminal H-T-H (red) and a 7 bladed β-propeller (green). Unlike DDB2, Raf1 contains an additional domain (blue) of unknown function. (TIF) [file pgen.1002499.s002.tif]

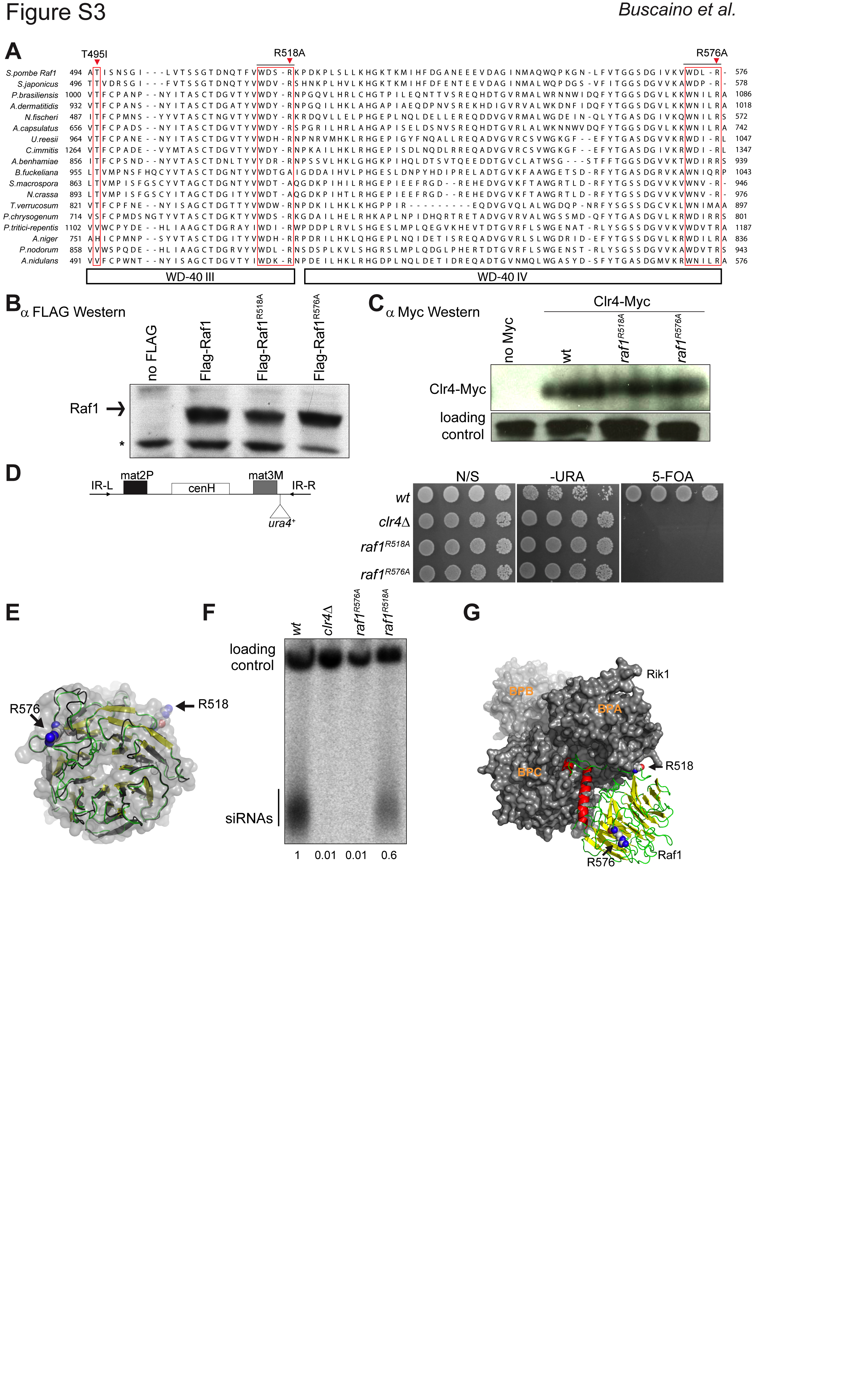

Supplement: Figure S3 — Raf1 contains two WDxR motifs important for protein function. (A) Representative multiple sequence alignment of the C-terminal WD-40 repeats of Raf1 in homologous proteins from fungi. Mutated residues mentioned in the text (T495I, R518A, R576A) are highlighted. (B) Western analyses of untagged, FLAG-Raf1, FLAG-Raf1R518A and FLAG-Raf1R576A from whole cell extracts. Wild-type and mutant Raf1 proteins (arrow) are expressed at similar levels. Asterisk *: indicates cross-reacting band which serves as a loading control. (C) Western analyses of untagged and Clr4-Myc in wild-type, raf1-R518A and raf1-R576A whole cell extracts. Loading control: Bip1. (D) Assay for silencing at mat3-M:ura4 +. Plates are non-selective (N/S), lacking uracil (-URA) or supplemented with 5-FOA. Loss of silencing results in growth on -URA and loss of resistance to 5-FOA. (E) Top view of Raf1 β-propeller ring. Residues R518 and R576 are highlighted. (F) Northern: centromeric siRNAs in wild-type, clr4Δ, raf1-R518A and raf1-R576A cells. Loading control: snoRNA58. (G) Rik1-Raf1 model showing the position of residues R518 and R576. (TIF) [file pgen.1002499.s003.tif]

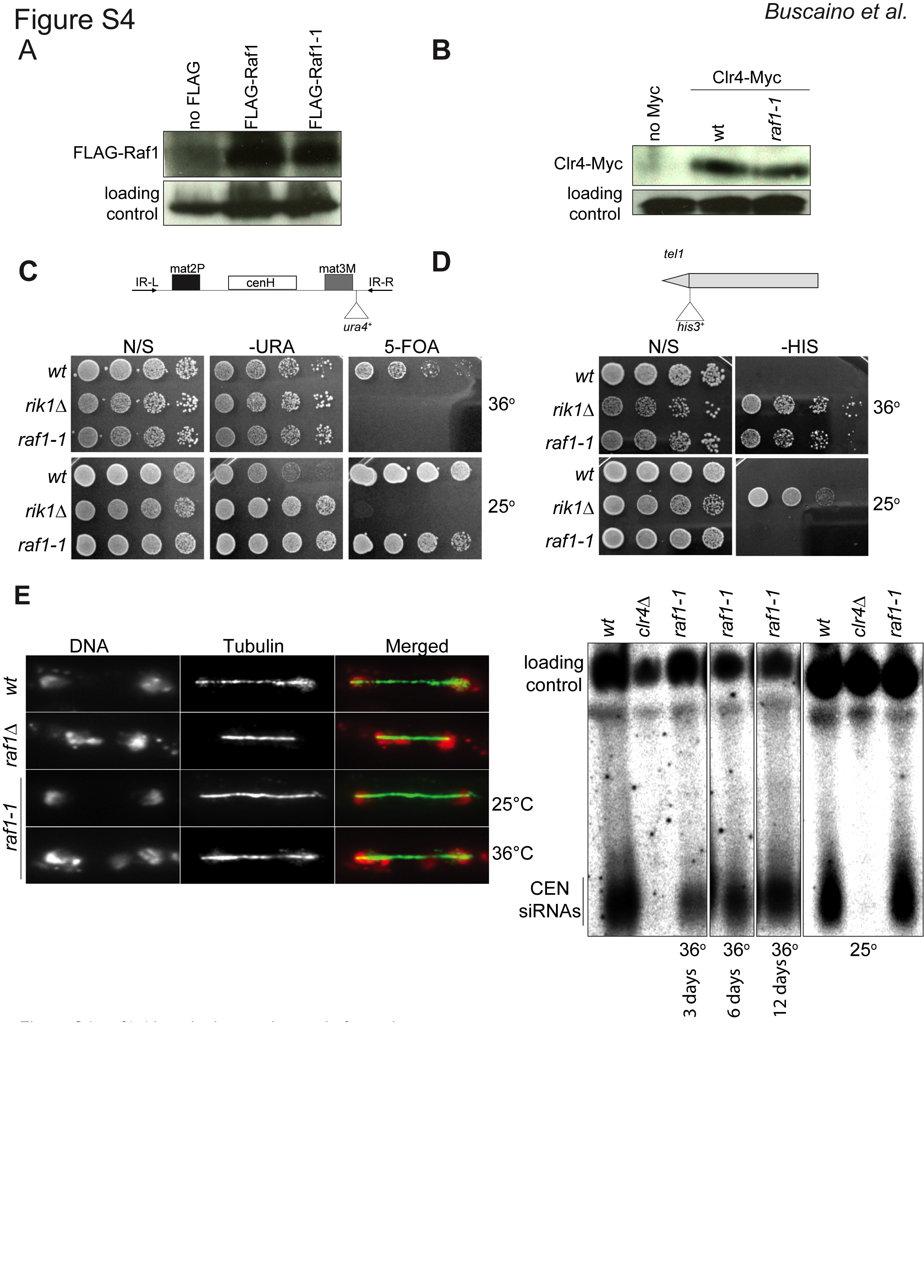

Supplement: Figure S4 — raf1-1 impairs heterochromatin formation. (A) Western analyses of untagged, FLAG-Raf1 and FLAG-Raf1-1 from whole cell extracts. (B) Western analyses of Clr4-Myc in wild-type and raf1-1 whole cell extracts. A strain not expressing Clr4-Myc (no Myc) is included as a control. Loading control: Bip1. Cells were grown at 36°C. (C) Assay for silencing at mat3-M:ura4 +. Plates are non-selective (N/S), lacking uracil (-URA) or supplemented with 5-FOA. Loss of silencing results in growth on -URA and loss of resistance to 5-FOA. (D) Assay for silencing at tel1L:his3 +. Plates are non-selective (N/S) and lacking histidine (-HIS). Loss of silencing results in growth on –HIS. (E) Lagging chromosomes in anaphase wild-type and raf1-1 cells at 25°C or 36°C. Representative images of fixed cells with DAPI (red) and anti-tubulin (green). (F) Northern: centromeric siRNAs in wild-type, clr4Δ, raf1-1 cells. Cells were shifted from 25°C to 36°C for the indicated period of time (3, 6 and 12 days resulted in 36, 72 and 144 divisions, respectively, at the restrictive temperature). Loading control: snoRNA58. (TIF) [file pgen.1002499.s004.tif]

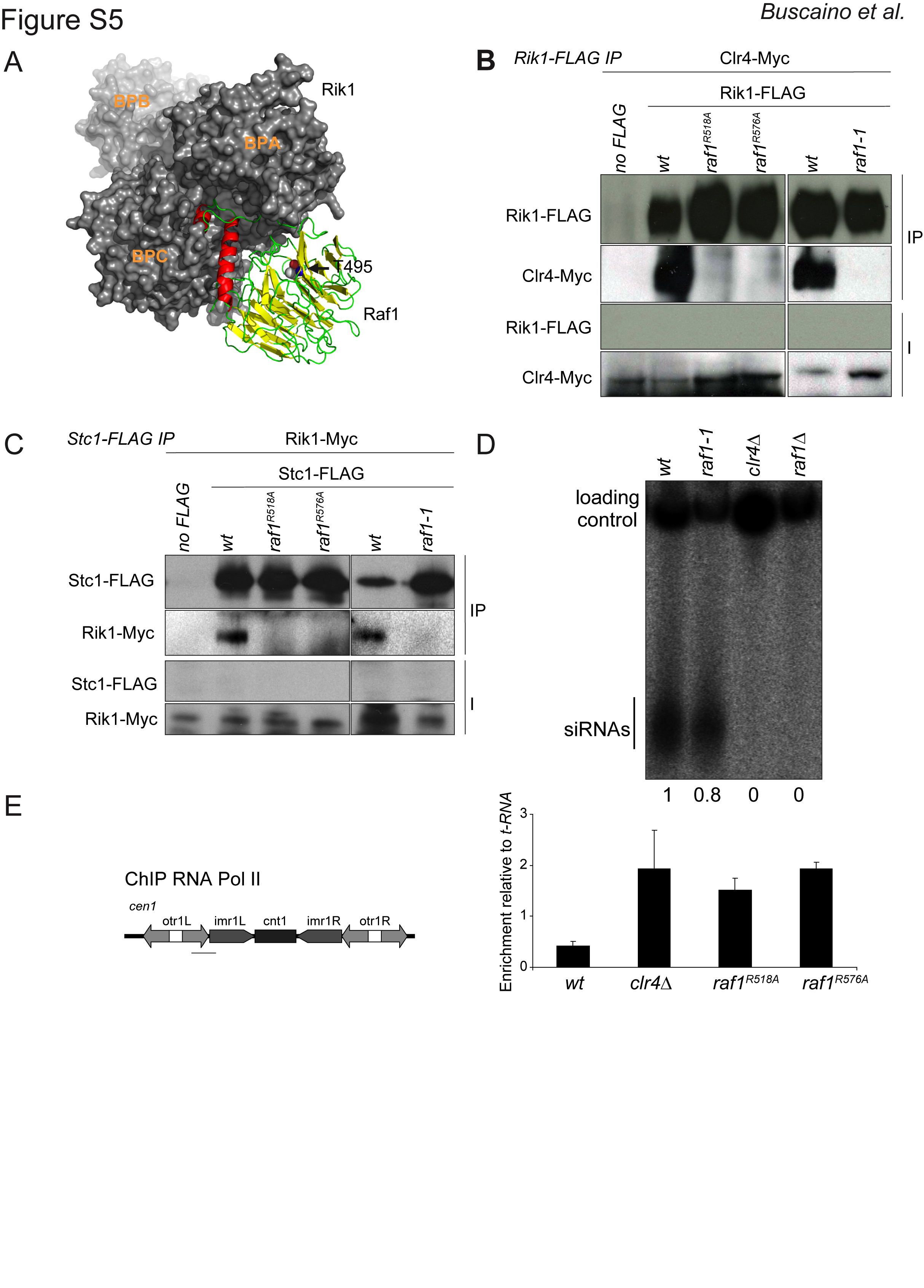

Supplement: Figure S5 — raf1-1, raf1-R518A and raf1-R576A disrupts CLRC and heterochromatin integrity. (A) Rik1-Raf1 model showing the position of the T495I mutatedresidue in raf1-1. (B) Westerns of Rik1-FLAG IP analysed for Clr4-Myc in wild-type, raf1-R518A, raf1-R576A and raf1-1 cells. Right: cells grown at 36°C. (C) Westerns of Stc1-FLAG IP analysed for Rik1-Myc in wild-type, raf1-R518A, raf1-R576A and raf1-1 cells. Right: cells grown at 36°C. (D) Northern: centromeric siRNAs in wild-type, raf1-1, clr4Δ, and raf1Δ cells. Cells grown at 36°C. Loading control: snoRNA58. (E) RNAPII ChIP in wild-type, clr4Δ, raf1-R518A and raf1-R576A cells. Diagram (left) indicates position of cen1 primers used (grey bar). RNAPII enrichment (right) was analysed by qPCR relative to tRNA gene primers. Error bar SD. (TIF) [file pgen.1002499.s005.tif]
