# Supplementary material for: Raf1 Is a DCAF for the Rik1 DDB1-Like Protein and Has Separable Roles in siRNA Generation and Chromatin Modification
Source: PLoS Genet. 2012 Feb 2;8(2):e1002499. doi: 10.1371/journal.pgen.1002499 (PMC3271066; doi:10.1371/journal.pgen.1002499)
Supplement: Table S1 — List of strains used in this study. (DOC) [file pgen.1002499.s006.doc]

|  | **Table S1. List of strains used in this study** | Relative to Figure |
| --- | --- | --- |
| FY 14359 | *h^-^ GFP-raf1::ura4^+^ leu1-32 ura4-D18* | 1 |
| FY 14669 | *h^+^ rik1-FLAG:NatMX6 GFP-raf1:ura4^+^ ade6-210 leu1-32 ura4-D18 otr1R(dg-glu)Sph1:ade6^+^* | 1 |
| FY 15928 | *Mat1Msmto 3XFlag-Raf1:KanMX6 otr1R(Sph1):ura4^+^ leu1-32 his2 ura4DS/E ade6-210* | 1 |
| FY 1180 | *h^+^ otr1R(dg-glu)Sph1:ade6^+^ ade6-210 leu1-32 ura4-D18* | 1; 2; 3 |
| FY 1181 | *h^-^ otr1R(dg-glu)Sph1:ade6^+^  ade6-210 leu1-32 ura4-D18* | 1; 2; 3 |
| FY 16630 | *h^90^ FLAG-raf1-R518A:NatMX6 otr1R(dg-glu)Sph1:ade6^+^ his3/his2* | 2 |
| FY 16671 | *h^90^ FLAG-raf1-R576A:NatMX6 otr1R(dg-glu)Sph1:ade6^+^ his3/his2* | 2 |
| FY 10554 | *h^+^ clr4::HphMX6 otr1R(dg-glu)Sph1:ade6^+^ his3D* | 2; 3; 4 |
| FY 11525 | *h^-^ raf1-1 otr1R(dg-glu)Sph1:ade6^+^ leu1-32 ura4-D18 ade6-210* | 3; 4 |
| FY 8137 | *h^-^ raf1::KanMX6 leu1-32 ade6-210 ura4-D18 his3D1* | 3; S4 |
| FY 511 | *h^90^ mat3-M::ura4^+^ ade6-216 leu1-32 ura4-D* | 4; S3; S4 |
| FY 9299 | *h^90^ dcr1::NatMX6 mat3-M::ura4^+^ ura4DS/E* | 4 |
| FY 9932 | *h^-^ ago1::3xFlag-ago1:KanMX6 ade6-210 arg3D his3D leu1-32 ura4-D18* | 4 |
| FY 15086 | *h^90^ raf1-1 mat3M::ura4^+^ otr1R(dg-glu)Sph1:ade6^+^ ade6216/ade6-210* | 4 |
| FY 15881 | *h^-^ ago1::3xFlag-ago1:KanMX6 raf1-1:KanMX6 ade6-210 otr1R(dg-glu)Sph1:ade6^+^ (?)* | 4 |
| FY 1645 | *h^+^ ade6-210 arg3-D4 his3-D1 leu1-32 ura4-D18* | 5 |
| FY 1646 | *h^-^ ade6-210 arg3-D4 his3-D1 leu1-32 ura4-D18* | 5 |
| FY 8635 | *h^+^ Nmyc-clr4^+^ otr1R(dg-glu)Sph1:ade6^+^ ade6-210 leu1-32 ura4D18* | 5 |
| FY 9733 | *h^90^ rik1-3Flag:NatMX6 otr1R(dg-glu)Sph1:ade6^+^ ade6-210* | 5 |
| FY 15550 | *h^+^ rik1-Flag:NatMX6 Myc-clr4 otr1R(dg-glu)Sph1:ade6^+^* | 5 |
| FY 15646 | *h^+/-^ rik1-FLAG:NatMX6 MycClr4 raf1-1 otr1R(dg-glu)Sph1:ade6^+^* | 5 |
| FY 15875 | *h^-^ raf2-FLAG:NatMX6 Myc-Clr4 raf1-1:KanMX6 otr1R(dg-glu)Sph1:ade6^+^ (?)* | 5 |
| FY 16766 | *h^+^ FLAG-raf1:NatMX6 otr1R(dg-glu)Sph1:ade6^+^ his3/his2* | 5 |
| FY 16818 | *h^90^ rik1^+^-13myc-KanMX6 FLAG-raf1^+-^NatMX6* | 5 |
| FY 16822 | *h^-^ rik1^+^-13myc-KanMX6 FLAG-raf1-1:NatMX6* | 5 |
| FY 17035 | *h^-^ raf2^+-^-TAP:KanMX6 FLAG-raf1-1:NatMX6* | 5 |
| FY 17038 | *h^+^ raf2^+-^-TAP:KanMX6 FLAG-raf1R576A-NatMX6 otr1R(dg-glu)Sph1:ade6^+^ ade6-216/210 his3/his2 o* | 5 |
| FY 17062 | *h^-^ raf2^+^-TAP:KanMX6 FLAG-raf1R518A-NatMX6 otr1R(dg-glu)Sph1:ade6^+^ ade6-216/210 his3/his2* | 5 |
| FY 17066 | *h^+/-^ raf2^+^-TAP:KanMX6 FLAG-raf1:NatMX6 otr1R(dg-glu)Sph1:ade6^+^(?) imR(Nco):ura4^+^(?) ade6210/216 his3/his2* | 5 |
| FY 16266 | *Mat1Msmto FLAG-raf1-1:NatMX6 otr1R(Sph1):ura4^+^ leu1-32 his2 ura4DS/E ade6-210* | 5 |
| FY 16566 | *h^+/-^ FLAG-raf1^+^:KanMX6 otr1R(dg-glu)Sph1:ade6^+^ his3/his2* | 5 |
| FY 17813 | *h^+/-^ rik1-FLAG:NaMX6 Myc-clr4^+^* | 5 |
| FY 17814 | *h^+/-^ FLAG-raf1-1:NatMX6 Myc-clr4^+^* | 5 |
| FY 17815 | *h^+/-^ FLAG-raf1-R518A:NatMX6 Myc-clr4^+^* | 5 |
| FY 17816 | *h^+/-^ FLAG-raf1-R576A:NatMx6 Myc-clr4^+^* | 5 |
| FY 8120 | *h^90^ rik1^+^-13myc-KanMX6 Kint2::ura4^+^ ade6-M216 his2 leu1-32 ura4-DSE* | 5 |
| FY 15885 | *h^-^ lid2^+^-TAP:ura4^+^ ura4-D18 leu1-32 ade6-210 his3D* | S1 |
| FY 16023 | *h^-^ FLAG-rik1:NatMX6 lid2^+^-TAP:ura4^+^* | S1 |
| FY 16773 | *h^90^ FLAG-raf1-R518A:NatMX6 mat3M::ura4^+^ otr1R(Sph1)::ade6^+^ (?)* | S3 |
| FY 16775 | *h^90^ FLAG-raf1-R576A:NatMX6 otr1R(dg-glu)Sph1:ade6^+^ (?)mat3M::ura4^+^* | S3 |
| FY 1051 | *h^90^ rik1::LEU2 mat3M::ura4^+^ ade6-210 his1-102 leu1-32 ura4DS/E* | S4 |
| FY 2117 | *h^+^ TEL1L-his3^+^ ade6-210 his3-D1 leu1-32 ura4-D18* | S4 |
| FY 2482 | *hA rik1::LEU2 otr1R(dg-glu)Sph1:ade6^+^ TEL1L-his3^+^ TEL2L-ura4^+^ ade6-210 arg3-D4 his3-D1 leu1-32 ura4-DS/E* | S4 |
| FY 12327 | *h? raf1-1 otr1R(dg-glu)Sph1:ade6^+^ TEL1L-his3^+^ leu1-32 ade6-210 ura4-D18 his3D* | S4 |
| FY 15550 | *h^+^ rik1-FLAG:NatMX6 Myc-clr4^+^ otr1R(dg-glu)Sph1:ade6^+^* | S3;S4 |
| FY 15646 | *h^+/-^ rik1-FLAG:NatMX6 Myc-clr4^+^ raf1-1 otr1R(dg-glu)Sph1:ade6^+^* | S4 |
| FY 17643 | *h^+/-^ rik1-FLAG:NtaMX6 Myc-clr4^+^ raf1-R576A:KanMX6* | S3 |
| FY 17645 | *h^+/-^ rik1-FLAG:NatMX6 Myc-clr4^+^ raf1-R518A:NatMX6* | S3 |
| FY 14599 | *h^+/-^ stc1-FLAG:NatMX6 rik1^+^-13myc-KanMX6 ade6-210/216 leu1-32 ura4-D18/DSE* | S5 |
| FY 17451 | *h^+/-^ stc1-3XFLAG:HphMX6 rik1^+^-13myc-Kan raf1-1:NatMX6* | S5 |
| FY 17453 | *h^+/-^ stc1-3XFLAG:HphMX6 rik1^+^-13myc-Kan raf1-R518A:NatMX6* | S5 |
| FY 17642 | *h^+/-^ stc1-3XFLAG:HphMX6 rik1^+^-13myc-Kan raf1-R576A:NatMX6* | S5 |
