# Supplementary material for: Raf1 Is a DCAF for the Rik1 DDB1-Like Protein and Has Separable Roles in siRNA Generation and Chromatin Modification
Source: PLoS Genet. 2012 Feb 2;8(2):e1002499. doi: 10.1371/journal.pgen.1002499 (PMC3271066; doi:10.1371/journal.pgen.1002499)
Supplement: Table S2 — List of primers used in this study. (DOC) [file pgen.1002499.s007.doc]

| **Table S2 : List of primers used in this study** | |
| --- | --- |
| **qPCR** |  |
| q_cen(dg)_FOR | AATTGTGGTGGTGTGGTAATAC |
| q_cen(dg)_REV | GGGTTCATCGTTTCCATTCAG |
| dg_rev3 | TTTGCAACCCACATATCGG |
| DM566 | TTATTGATGGCGAAGCTAGATCCG |
| q_act_FOR | GGTTTCGCTGGAGATGATG |
| q_act_REV | ATACCACGCTTGCTTTGAG |
| WA33 (fbp) | AATGACAATTCCCCACTAGCC |
| WA34 (fbp) | ACTTCAGCTAGGATTCACCTGG |
| qtRNA Gly Fw | AATGCTTTGGCCGGGAATCGAA |
| qtRNA Gly Rev | CATTAAGCTTTGGTGGTTTAG |
|  |  |
| **Mutagenesis** |  |
| raf1R518A | TTTGGGATTCGGCTAAACCAGATAA |
| raf1R518A_rev | TTATCTGGTTTAGCCGAATCCCAAA |
| raf1R576A | TTTGGGATTTGGCATTGAACAATCC |
| raf1R576A_rev | GGATTGTTCAATGCCAAATCCCAAA |
|  |  |
| **Northern probes** |  |
| censiRNAs |  |
| IK8 | ATTCCTTTCTGAACCTCTCTGTTAT |
| IK9 | TTTGATGCCCATGTTCATTCCACTTG |
| IK10 | GGGAGTACATCATTCCTACTTCGATA |
| snR58 | GATGAAATTCAGAAGTCTAGCATC |
| Ing-dg-FOR | CTACTCTTCTCGATGATCCTG |
| Ing-dg-REV | GTAGTACGACGATGATGTGTTTTC |
|  |  |
