# Supplementary material for: Raf1 Is a DCAF for the Rik1 DDB1-Like Protein and Has Separable Roles in siRNA Generation and Chromatin Modification
Source: PLoS Genet. 2012 Feb 2;8(2):e1002499. doi: 10.1371/journal.pgen.1002499 (PMC3271066; doi:10.1371/journal.pgen.1002499)
Supplement: Text S1 — Supplementary experimental procedures. (DOC) [file pgen.1002499.s008.doc]

**Supplementary Experimental Procedures**

**Plasmids and strain Construction**

Standard procedures were used for bacterial and fission yeast growth and genetic

manipulations *. S. pombe* strains used in this study are described in Table S1. Primer sequences are listed in Table S2.

*Raf1-R518A* and *Raf1-R576A* mutations were generated by fusion PCR using oligos raf1R518A, raf1R18Arev, raf1R576A and raf1R576Arev listed in Table S2 . The *raf1-R518A-NatMX6* or *raf1-R576A-NatMX6* fusion PCR products were transformed by electroporation in the FY16566 strain. Correct integrants were selected by replacement of the *KanMX6* cassette with the *NatMX6* cassette and sequencing of the raf1 gene.

*Raf1-1* allele was isolated in a random UV mutagenesis genetic screen. FY 1181 cells were spread on YES plates lacking adenine, irradiated with 15000 μJ (around 50% killing) and incubated at 36°C for 5-7 days. Fast-growing colonies were picked and tested for thermosensitivity of silencing at *otr1R(Sph):ade6^+^* and for supersensitivity to TBZ. Mutants were backcrossed at least three times. *Raf1-1* mutation was identified by non-complementation and sequencing of the raf1 gene. *FLAG-raf1-1:KanR* and *FLAG-raf1-1:NatR* were generated by fusion PCR and integration .

Yeast two-hybrid vectors were generated by PCR amplification of the Rik1 ORF with primers bearing EcoR1 and BamH1 sites, and of the Raf1 ORF with primers containing NcoI and Xma1 sites, or Xma1 and Cla1 sites. Purified Rik1 PCR products were digested and cloned into EcoR1/BamH1 digested pGADT7 or pGBKT7; purified Raf1 PCR products were digested and cloned into NcoI/Xma1 digested pGBKT7, or Xma1/Cla1 digested pGADT7 (Clontech ‘matchmaker’ system). Plasmids pGAD-Raf1R518A and pGAD-Raf1R576A were generated with QuikChange XL Site-Directed Mutagenesis Kit (Stratagene #200516).

**Modelling**

The homology model of Raf1 was created with the program Modeller (9v8) using the structure of DDB2 (PDB code 3EI4) . Iterative rounds of alignment adjustment of the two protein sequences and model building were attempted until the Modeller scores (diagnostic of model quality) were optimised. The GA341 score is designed to differentiate between good and poor models. It is calculated by taking into account the "compactness" of the model (i.e. the surface area to volume ratio), the Z-score (based on the energy of the model) and the percentage identity between the sequences. In the case of WD40 proteins, their toroidal shape increases their surface area to volume ratio, reducing the "compactness" rating used by Modeller in the GA341 calculation. The sequence identity between the two proteins is also low, which further reduces this score. Despite these unavoidable factors, the best model produced by Modeller gained a GA341 score of 0.6, which is generally deemed to be the acceptable cutoff for determining that a model is accurate. The other main score output by Modeller, the DOPE (Discrete Optimized Protein Energy) score, ranks highest the same model that the GS341 score favours, supporting the identification of this model as the most accurate from the ensemble produced. The homology model of Rik1 was created with the program Modeller (9v8) using 5 templates (PDB code: 2B5L; 317N; 3189; 318C; 3E0C) . The generated model (85.2) had the following model scores: RMSD: 1.536; MolPDF: 453.98; DOPE: -11385.

**Sequence Alignment**

The sequence alignment of Figure S3A was generated using BLAST ([http://blast.ncbi.nlm.nih.gov](http://blast.ncbi.nlm.nih.gov/)) and Jalview alignment editor ([www.**jalview**.org/](http://www.jalview.org/)). To generate the alignment the following proteins were used:

S.japonicus: XP_002172025.1; P.brasiliensis: EEH43419.1; A.dermatitidis: XP_002620382.1; N. fischeri: XP_001266102.1; A. capsulatus: EEH02942.1; U. reesii: XP_002544562.1; C.immitis : XP_001242421.1; A. benhamiae : EFE34496.1; B. fuckeliana :XP_001547061.1; S. macrospore: CBI53136.1; N. crassa: XP_956278.2; T. verrucosum : EFE41806.1; P. chrysogenum: XP_002565067.1; P.tritici-repentis: XP_001933855.1; A. niger: XP_001393992.1; A. nidulans: XP_681551.1.

**ChIP: Chromatin Immunoprecipitation**

ChIP was performed as described with the following modifications. Cells were fixed in 1% PFA/15 min for H3K9me2 ChIP or in 1% PFA/20 min for RNA Polymerase II ChIP. For ChIP analyses of *raf1-R518A* and *raf1-R576A* strains*,* cells were grown at 32^o^C. For ChIP analyses of *raf1-1* strain*,* cells were kept at the indicated temperature (25^o^C, 32^o^C or 36^o^C) for at least 96 hours. One microliter of monoclonal H3K9me2 antibody (m5.1.1), two microlitres of anti-FLAG M2 monoclonal antibody (Sigma, F1804) or five microlitres of RNA Polymerase II 8WG16 antibody (COVANCE, MMS-126R) was used per ChIP. Competitive duplex PCR was performed to analyse ChIP samples using oligonucleotides specific to the regions of interest and to the control gene *fbp1* (Table S2). Real-time PCR (qPCR) was performed using the LightCycler 480 SYBR Green I Master (Roche) on a LightCycler 480 Instrument (Roche). qPCR analysis primers are in Table S2. Relative enrichments were calculated as the ratio of product of interest to control product (*act1*^+^ or *tRNA*) in IP over input. Histograms represent data from three biological replicates analysed in parallel

**Immunoaffinity Purification (IP)**

Immunoaffinity purifications for LC-MS/MS analysis were performed as described , with the following modifications: 5g of cells were resuspended in ice-cold lysis buffer (50mM Hepes pH7.5, 150mM KCl, 0.1% NP40). Immunoprecipitation was performed using proteinG Dynabeads resin (Life Technologies) coupled to anti-FLAG M2 antibody (Sigma, F1804) for 15 min. The IP'd material was treated with 500U Benzonase, washed, subjected to on-bead Tryptic digestion, and prepared for LC-MS/MS analysis as described previously .

Co-IPs for Western analysis were performed on 2g of cells as above but for 1 hr. IP’d material was washed four times with ice-cold lysis buffer (50mM Hepes pH7.5, 150mM KCl, 0.1% NP40), resuspended in SDS sample buffer, and analysed by SDS-PAGE. For tandem affinity purification (TAP)-tagged strains, Dynabeads coupled to IgG were used (gift from K. Hardwick). For Western analysis the following antibodies were used: anti-FLAG M2 (Sigma, F1804), anti-HA 12CA5 (gift from K. Samejima), anti-GFP (gift from K. Hardwick) and anti-myc9E10 (Covance), all at 1:1000.

**Cytology**

Cells were fixed with 3.7% PFA/10 min, plus 0.05% glutaraldehyde for tubulin staining. Antibodies used were TAT1 anti-tubulin 1:15 (gift from K. Gull), anti-Cnp1 and anti-Swi6 1:1000 . Alexa Fluor 594- and 488-coupled secondary antibodies were used at 1:1000 (*Life Technologies*).

**RNA analysis**

Northern analysis of long non-coding centromeric transcripts and centromeric siRNAs were performed as described previously . RNA probes are listed in Table S2. For Ago1-associated siRNA preparation, 10g of cells were lysed in the presence of liquid nitrogen using a mortar grinder (Retsch) for 30 minutes. Extracts were prepared by dilution of the crushed cells in 20ml lysis buffer (50 mM Hepes-NaOH (pH 7.5), 150 mM NaCl, 1 mM MgCl2, 0.1% NP-40, 5mM DTT, 1x Roche EDTA-free protease inhibitors cocktail, 0.5mM PMSF, 1/100 super RNAsin (Ambion), and filtration through GD/X 1.6 μm filter (Whatman). Immunoprecipitations were performed using 20 micrograms of M2 anti-Flag antibody (Sigma F1804) coupled to 4 microlitres of proteinG Dynabeads resin (*Life Technologies*) for 15 minutes at 4 °C. RNA samples bound to Dynabeads were washed once with lysis buffer and once with lysis buffer with 2 mM MgCl2 added, treated with 200 ng/ml proteinase K (Sigma), extracted first with phenol/chloroform and finally with chloroform. RNA was precipitated with ethanol, sodium acetate, and glycogen.

**References**

1. Moreno S, Klar A, Nurse P (1991) Molecular genetic analysis of fission yeast Schizosaccharomyces pombe. Methods Enzymol 194: 795-823.

2. Yu JH, Hamari Z, Han KH, Seo JA, Reyes-Dominguez Y, et al. (2004) Double-joint PCR: a PCR-based molecular tool for gene manipulations in filamentous fungi. Fungal Genet Biol 41: 973-981.

3. Scrima A, Konickova R, Czyzewski BK, Kawasaki Y, Jeffrey PD, et al. (2008) Structural basis of UV DNA-damage recognition by the DDB1-DDB2 complex. Cell 135: 1213-1223.

4. Li T, Chen X, Garbutt KC, Zhou P, Zheng N (2006) Structure of DDB1 in complex with a paramyxovirus V protein: viral hijack of a propeller cluster in ubiquitin ligase. Cell 124: 105-117.

5. Li T, Robert EI, van Breugel PC, Strubin M, Zheng N (2010) A promiscuous alpha-helical motif anchors viral hijackers and substrate receptors to the CUL4-DDB1 ubiquitin ligase machinery. Nat Struct Mol Biol 17: 105-111.

6. Pidoux A, Mellone B, Allshire R (2004) Analysis of chromatin in fission yeast. Methods 33: 252-259.

7. Oeffinger M, Wei KE, Rogers R, DeGrasse JA, Chait BT, et al. (2007) Comprehensive analysis of diverse ribonucleoprotein complexes. Nat Methods 4: 951-956.

8. Bayne EH, Portoso M, Kagansky A, Kos-Braun IC, Urano T, et al. (2008) Splicing factors facilitate RNAi-directed silencing in fission yeast. Science 322: 602-606.

9. Ekwall K, Javerzat JP, Lorentz A, Schmidt H, Cranston G, et al. (1995) The chromodomain protein Swi6: a key component at fission yeast centromeres. Science 269: 1429-1431.
